# Autonomous Self-Healing Strategy for Stable Sodium-Ion Battery: A Case Study of Black Phosphorus Anodes

**DOI:** 10.1021/acsami.0c22464

**Published:** 2021-03-15

**Authors:** D. Callegari, S. Colombi, A. Nitti, C. Simari, I. Nicotera, C. Ferrara, P. Mustarelli, D. Pasini, E. Quartarone

**Affiliations:** † Department of Chemistry and INSTM, University of Pavia, Via Taramelli 16, 27100 Pavia, Italy; ‡ Department of Chemistry and Chemical Technologies, Università Della Calabria, Via Pietro Bucci, 87036 Arcavacata di Rende, Cs Italy; § Department of Materials Science, University of Milano Bicocca, Via Cozzi 55, 20125 Milano, Italy; ∥ National Reference Centre for Electrochemical Energy Storage (GISEL)—INSTM, Via G. Giusti 9, 50121 Firenze Italy

**Keywords:** self-healing binder, supramolecular polymers, quadruple hydrogen bonding, black phosphorus, Na-ion batteries

## Abstract

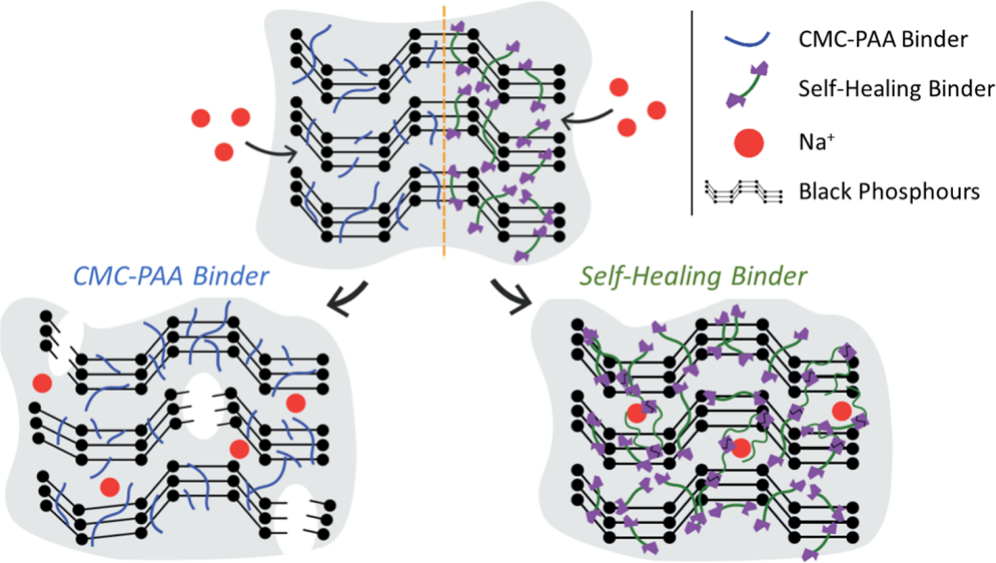

Autonomic self-healing (SH), namely, the ability to repair damages from mechanical stress spontaneously, is polarizing attention in the field of new-generation electrochemical devices. This property is highly attractive to enhance the durability of rechargeable Li-ion batteries (LIBs) or Na-ion batteries (SIBs), where high-performing anode active materials (silicon, phosphorus, *etc.*) are strongly affected by volume expansion and phase changes upon ion insertion. Here, we applied a SH strategy, based on the dynamic quadruple hydrogen bonding, to nanosized black phosphorus (BP) anodes for Na-ion cells. The goal is to overcome drastic capacity decay and short lifetime, resulting from mechanical damages induced by the volumetric expansion/contraction upon sodiation/desodiation. Specifically, we developed novel ureidopyrimidinone (UPy)-telechelic systems and related blends with poly(ethylene oxide) as novel and green binders alternative to the more conventional ones, such as polyacrylic acid and carboxymethylcellulose, which are typically used in SIBs. BP anodes show impressively improved (more than 6 times) capacity retention when employing the new SH polymeric blend. In particular, the SH electrode still works at a current density higher than 3.5 A g^–1^, whereas the standard BP electrode exhibits very poor performances already at current densities lower than 0.5 A g^–1^. This is the result of better adhesion, buffering properties, and spontaneous damage reparation.

## 1 Introduction

The future next-generation metal (M)-ion batteries [M = Li-ion batteries (LIBs) and Na-ion batteries (SIBs)] need to be improved in terms of enhanced durability, lower cost per stored energy, and sustainability. Some of the most crucial issues which are detrimental on the battery performances are related to the physical chemistry of the electroactive materials selected as anode components.^1^ The electrochemical processes taking place in the anode compartment, in fact, involve dimensional/structural evolutions which cause degeneration, damage, and serious cycling failure. This is particular evident in anodes based on silicon^2^ or phosphorus,^3^ which normally undergo huge volumetric expansion/contraction upon full Li or Na insertion/deinsertion, thereby forcing large material strains. This results in electrode mechanical fracture, leading to cracking, pulverization, loss of electrical contact with the current collector, and even uncontrolled solid electrolyte interphase (SEI) growth. All the previously reported Si- and P-based anodes showed capacity fading higher than 70–80% over the first 10–20 cycles.^2,3^

Several approaches were discussed in the literature to enhance the durability of Si and P anodes in order to exploit their high theoretical capacity, such as the use of nanostructures or composites with carbon acting as a buffer agent.^2−6^ Very recently, an innovative strategy to overcome such concerns is emerging, which finds inspiration from biology and specifically from the capability of some materials/tissues to self-heal or recover from a physical damage to prevent them from the loss of their vital functions.^7^ In this frame, polymers are particularly appealing because of their good self-healing (SH) capabilities involving either physical (*e.g.*, interchain diffusion, phase-separated morphology, and shape memory effects) or chemical (incorporation of covalent, free radical, or supramolecular dynamic bonds) features, which employ a synergistic combination of hydrogen bonds (HBs), van der Waals forces, and chemical reactions to repair microdamages, autonomously or even upon external triggering by variation of pH, temperature, pressure, and so forth.^7−11^ SH polymers (SHPs) are finding applications as active components in the improvement of electrochemical energy storage devices, including LIBs. For this specific application, several possible recovering strategies were explored, including different healing mechanisms (physical *vs* chemical), processes, and materials.^12−14^

In order to develop new electrodes with high energy density and less irreversible mechanical fractures, SPHs were added chiefly to microparticles of silicon, both pure and as composites with carbon.^15−19^ In this frame, SHPs acted as a new concept of binder, properly designed to be multifunctional as (i) a mechanical stabilizer, (ii) a structural support, (iii) wettable by the electrolyte, and also (iv) capable of recovering the physical damage caused by the structural changes.^20^ Basically, SPHs were used following two different approaches: (i) as a thin soft layer of randomly branched hydrogen-bonded strands coating the Si electrode^18^ or (ii) as a conventional binder surrounding the electrode particles and binding them to the current collector.^21^ In both cases, very promising results were obtained in terms of cracks and damages recover upon cycling and, consequently, significant improvements in terms of anode capacity retention.^14,19,22^

Among the possible SH mechanisms, such as covalent bonding, supramolecular chemistry, ion–ion interaction, π–π-stacking, and dynamic H-bonding, the latter one received specific attention in the case of battery application.^14,22−24^ In fact, even though H-bonds among neutral systems are not particularly strong, they confer significant mechanical strength due to the high directionality and affinity.^7^ This is well evident, especially if multiple H-bonds are combined into a functional unit, for example, ureidopyrimidinone (UPy), which strongly associates with polymers potentially adaptable to the battery components (*e.g.*, polyethers). In this case, the mechanism is dynamic and requires high free volume and fluid-like systems which allow the orientation and approaching of the polymer chains, thus favoring the H–H interactions and consequently the SH mechanism. These supramolecular polymers exhibit low glass transition temperature, *T*_g_, resulting in soft systems with high segmental motion, easily modeled, but mechanically strong.^8,11^ Furthermore, an SHP based on dynamic bonding is capable of repeated and spontaneous healing without any external stimulus^11,25^ even at room temperature, as in the case of UPy-containing brush-like poly(ethylene glycol) (PEG) chains^24^ or upon gentle heating, as shown by maleimide–furan-based polymers.^26^

If silicon anodes have been widely investigated in terms of SH strategies, very few examples are to date reported about phosphorus but for a work employing inorganic anchoring units to protect the active material surface.^27^

In this paper, we describe, for the first time to the best of our knowledge, an autonomic SH strategy to improve the cycling lifetime of black phosphorus (BP) anodes for SIBs. To this aim, a novel H-bonding directed polymer is developed as an electrode binder with the specific role to repair the mechanical damages induced by the BP huge expansion and strain upon cycling. The system is based on PEG telechelic polymers decorated with UPy-chain end functionalities exhibiting the ability to self-recover at room temperature in the absence of external stimuli. The SH capability of the polymer is investigated by means of a multitechnique approach. Its effect on the BP anode electrochemical performances is demonstrated by comparing with similar electrodes including conventional binders, such as polyacrylic acid (PAA) and carboxymethylcellulose (CMC).

## 2 Materials and Methods

### 2.1 Materials

Compound UPy-NCO 1 was synthesized as reported elsewhere.^28^ Di-(OH)-terminated PEGs were purchased with different molecular weights (PEG_*n*_, *M*_W_ = 4000 Da, *n* = 91; *M*_W_ = 6000 Da, *n* = 136; *M*_W_ = 10,000 Da, *n* = 227; and *M*_W_ = 35,000 Da, *n* = 795) as monodisperse products from different commercial sources and were used as received. All other commercially available compounds were used as received.

### 2.2 Synthesis of UPyPEG_*n*_UPy (2–5) Polymers: PEG 2-(6-Isocyanato-hexilaminocarbonylamino)-6-metyl-4-[1*H*]-pyrimidinone

**Scheme 1:**
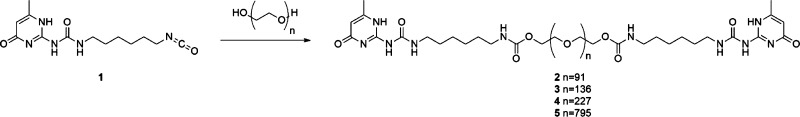
Synthesis by UPy-Terminated PEG Telechelics (UPyPEG_*n*_UPy) Developed in This Paper Reaction conditions: dry CHCl_3_, dibutyltin dilaurate (cat.), 60 °C, and 48 h.

The representative procedure for the synthesis of all the polymers 2–5, sketched in Scheme 1, is reported in detail for the specific polymer 5 (UPyPEG_795_UPy).

UPy-NCO 1 (166.4 mg, 0.568 mmol) was added to a solution of PEG_795_ (5 g, 0.143 mmol) in CHCl_3_ previously dried over molecular sieves (10 mL) in the presence of a catalytic amount of dibutyltin dilaurate (two drops). The resulting reaction mixture was stirred at 60 °C under inert atmosphere for 48 h. The mixture was cooled and filtered off to remove the exceeding UPy-NCO (compound 1). The last purification step was precipitation in hexane. The precipitate was recovered by filtration and washed plentifully with hexane, followed by drying under a reduced pressure to obtain UPyPEG_*n*_UPy as a white powder (4,9 g, 97%). ^1^H NMR of UPyPEG_795_UPy (200 MHz, CDCl_3_): δ 5.89 (s, 2H, CH=CCH_3_), 4.07 (d, *J* = 6.9 Hz, 4H, OCH_2_(CH_2_OCH_2_)CH_2_O), 4.02–3.17 (m, 2093H, OCH_2_(CH_2_OCH_2_)CH_2_O), 3.11 (m, 8H, NH(C=O)NHCH_2_ + CH_2_NH(C=O)O), 2.15 (s, 6H, CH_3_C=CH), 1.48 (m, 8H, NHCH_2_CH_2_ + CH_2_CH_2_NHC=O), 1.34 (m, 8H, CH_2_CH_2_CH_2_CH_2_CH_2_CH_2_). ^13^C NMR (75 MHz, CDCl_3_): δ = 172.8, 156.3, 154.4, 148.1, 106.4, 42.6, 39.5, 30.9, 29.1, 26.0, 19.9, 15.7.

### 2.3 Preparation of SH Blends of (UPyPEG_795_UPy)—PEO

Three blends of UPyPEG_795_UPy and PEO (300 kDa) were obtained by mixing UPyPEG_795_UPy and PEO in deionized water at different volume concentrations 40/60 (40–60), 50/50 (50–50), and 60/40 (60–40) (see Table 1). The final mixture was then cast on a Teflon foil and dried at 50 °C under vacuum in order to obtain homogeneous 110 μm-thick films.

**Table 1:** Glass Transition Temperatures, *T*_g_, Melting Enthalpies, Δ*H*_m,s_, and Crystalline Fraction, *X*_*c*_, (Estimated from ^1^H and ^13^C Solid-State NMR Spectra) of the Investigated Blends and Pure Components

	A/B (vol %)	*T*_g_ (°C)	Δ*H*_m,s_ (J g^–1^)	*X*_c_ (%) from ^1^H	*G*′ (MPa) as prepared	*G*′ (MPa) restored
UPyPEG_795_UPy (A)		–22.3	184.4			
PEO (B)		–25.6	198.6			
40–60	40/60	–26.7	152.9	24	9.8	9.6
50–50	50/50	–27.1	166.0	34	12.7	16.7
60–40	60/40	–32.7	136.8	30		

### 2.4 Preparation of BP Anodes

Two electrodes were fabricated, which differ for the binder component. The starting slurries were prepared through aqueous processing by mixing proper amounts of BP, carbon (Imerys Super C65), and the polymer binder with a 5/3/1 weight ratio. BP was synthesized *via* high-energy ball milling, as deeply described elsewhere.^6,29^ Raman spectra, SEM images, and XRD patterns of the as-prepared BP and the BP-C mixture used to prepare the anode slurry are reported in Figures S1–S3, respectively. Two different binders were used: (i) the SH UPyPEG_795_UPy—PEO blend (50–50) and (ii) a blend of Na-CMC and PAA (1/1 wt/wt). In detail, BP and carbon were initially ball milled for few minutes; in a typical process, the binder was dissolved in excess water, added to the two components, and successively mixed in a planetary ball mill at 200 rpm for 2 h, followed by a 10 min stop and other 2 h of milling in reverse direction. The solid content of the slurries was kept between 12% and 15 wt %. The resulting inks were then cast on an aluminum foil (UACJ, 15 μm thick) using a doctor blade to obtain a wet film with a thickness of 100 μm that was immediately dried under vacuum at 80 °C to avoid moisture and oxygen contamination. The anode was finally cut into 2 cm^2^ disks and stored in a glove box (MBraum, O_2_, H_2_O < 0.5 ppm) before the electrochemical measurements. In the case of electrodes based on the SH blend (50–50), two BP mass loadings were explored, ∼1.26 and ∼2.5 mg cm^–2^; in the electrode including CMC–PAA as a binder, the mass loading was 1.6 mg cm^–2^. Lower loadings were tested for the former system in our previous paper.^6^

### 2.5 Methods

Thermogravimetric analyses of the SH polymers were performed by heating aliquots of about 20 mg at 5 °C/min from room temperature up to 250 °C under a N_2_ atmosphere in a Pt crucible by means of a Q5000 thermogravimetric instrument (TA Instruments, USA). Differential scanning calorimetry (DSC) analyses were performed with a Q2000 instrument (TA Instruments, USA) by heating the samples (about 20 mg) from −80 to 150 °C at 5 °C/min under a N_2_ atmosphere in Al crucibles sealed in the glove box.

^1^H and ^13^C NMR high-resolution spectra were recorded on Bruker 300 and 400 MHz instruments and calibrated with the solvent residual proton signal. CHCl_3_ was dried using 4 Å molecular sieves.

Solid-state NMR measurements were acquired on a Bruker AVANCE III 400 MHz (9.4 T) equipped with a 4 mm probe at 27° ± 1 °C under MAS condition (10 kHz). ^1^H one-pulse data were collected after T_1_ determination to ensure the quantitative measurement condition with the use of 90^o^ pulse of 3 μs and 16 scans; T_1_ was evaluated with the use of a standard inversion recovery pulse sequence. For all the samples the same experimental conditions (contact time and decoupling scheme) were used. Rotors were filled with the membranes in the same quantity by cutting the membrane in macroscopic pieces (∼2 × 2 mm); this sample preparation was necessary to obtain stable MAS rotation and likely does not change the interchain interactions under investigation.

Dynamic mechanical analysis (DMA) measurements were performed on rectangular-shaped samples (35 mm × 10 mm), directly cut from the piece, by a Metravib DMA/25 equipped with a shear jaw for films. The frequency sweep experiments were carried out in the frequency range between 0.2 and 20 Hz at a constant strain of 0.004% from 20 to 60 °C every 10 °C. Temperature sweeps were performed at a heating rate of 2 °C, over a range between 20 and 60 °C, at a dynamic stress of amplitude 4 × 10^–3^ and a frequency of 1 Hz. For the stress–strain test, the sample was clamped on the tensile module with a separation of 10 mm. The speed rate was fixed at 0.2 mm min^–1^. Membrane’s thickness ranged between 100 and 110 μm.

The electrochemical characterization of the anodes was performed by means of galvanostatic cycling and electrochemical impedance spectroscopy (EIS) by using 2032-type coin cells assembled in an argon-filled glove box (H_2_O and O_2_ below 0.5 ppm) with Na metal both as a counter and reference electrode. Electrodes were separated with a *Whatman* glass fiber separator (GF/D) soaked in a 1.0 M solution of NaPF_6_ in EC/PC (50:50 vol %) with 5 wt % of NaTSFI and 2 wt % of FEC. The cells were cycled on a *Biologic* BCS 810 battery tester from 0.02 to 2 V at various C-rates (1C = 2596 mA h g^–1^). All the potentials reported refer to the Na^+^/Na couple. Rests of 48 h were typically imposed every six cycles in order to evaluate the SH effect on the cycling performances. The impedance on the cells was measured by means of EIS at room temperature by applying an AC voltage of 100 mV in the frequency range of 0.1 Hz to 1 MHz.

SEM analyses on the pristine anode and on post-mortem (both top-view and cross-section) were performed using a Tescan Mira3XMU microscope operated at 20 kV and equipped with an EDAX EDS analysis system. The samples were coated with a carbon thin film using a Cressington 208 carbon coater.

## 3 Results and Discussion

### 3.1 SH Binder: Material Design and Characterization

**Figure 1:**
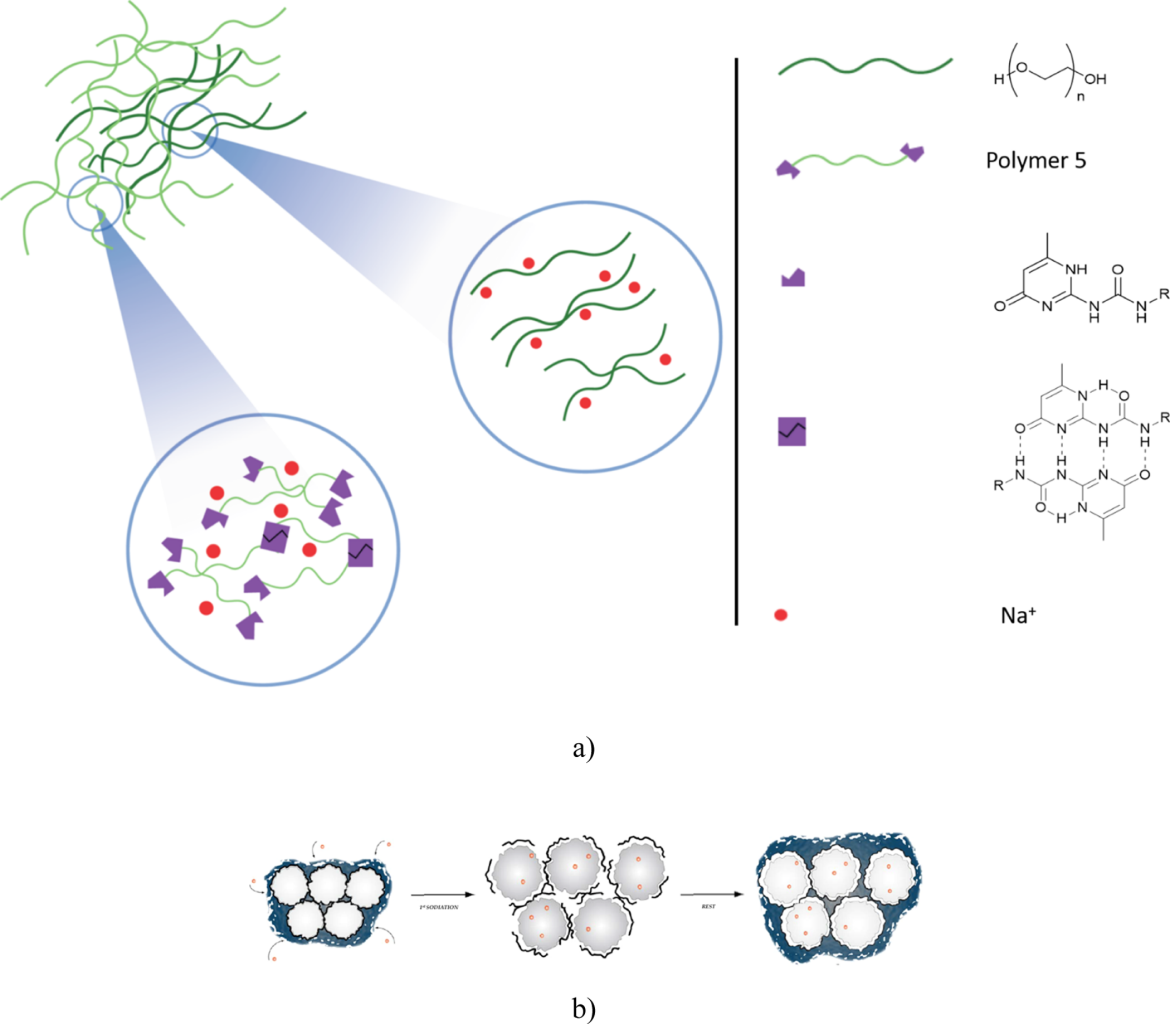
Design and working mechanism of the SH BP anode: (a) schematic representation of the binder and (b) expected functional reversible behavior of the UPyPEG_795_UPy—PEO blends. Red point: intercalating Na ions; black lines: repairing polymer backbones; and blue: electrode texture.

As stated before, we used a chemical approach to develop a new binder for anodes in SIBs capable of SH from physical damages upon long cycling and to mitigate the huge volume expansion of the BP anode. Figure 1 shows a naïve picture of the SH binder working with a dynamic hydrogen bonding mechanism boosted by the presence of two UPy functional groups in the backbone, which should promote a good adhesion among the BP particles themselves and also to the current collector. The SH polymer (UPyPEG_*n*_UPy) includes PEG units playing a dual role (i) to assist the Na ion transport within the anode and conferring ion-conducting properties to the binder and (ii) to decrease the charge transfer anode resistance, thus resulting in an enhanced electrochemical kinetics.^30^ The SH network (UPyPEG_*n*_UPy) was further blended with high-molecular weight (MW) polyethylene oxide (*M*_W_ = 300 kDa), which is physically and chemically affine, in order to achieve better free-standing properties and improve the dispersion of PB and carbon aggregates.

#### 3.1.1 SH UPyPEG_*n*_UPy Unit

2-Ureido-4[1*H*]-pyrimidone (UPy) containing supramolecular polymers were thoroughly discussed [see, for instance, refs^7,11,31^] in view of their excellent dynamic and supramolecular properties, including UPy-functionalized telechelics. Telechelics are the most classical of supramolecular polymers, where the highly directional physical interactions are applied by replacing some of the covalent bonds with supramolecular ones.^32^

Here, the quadruple hydrogen bonding group (UPy), by association *via* noncovalent interactions, endcaps a preformed short polymer chain, leading to a strong increase of the virtual molecular weight and to a concurrent improvement of its mechanical and rheological properties. Such end–end associations also result in a further enhancement of the SH capability of the supramolecular polymers due to a longer linear chain extension.^33,34^ The preformed central segment is typically given by a thermoplastic polymer such as polybutadiene, polystyrene, polyethylene, and so forth. Other polymeric chain segments for UPy-based telechelics, such as PEG, indeed more interesting for application in LIBs and SIBs, are less explored^35^

The SH UPyPEG_*n*_UPy polymers were synthetized as detailed in the Materials and Methods section (Scheme 1) by the covalent anchoring of isocyanate-functionalized UPy synthons to the terminal OH groups of PEG_*n*_ (1:2 M ratio). In principle, the reaction is simple, but the process must be optimized to minimize the degree of PEG monofunctionalization. Indeed, monofunctional species can work as chain stoppers in the supramolecular polymerization of the difunctional UPy telechelics. The experimental parameters favoring the complete difunctionalization and then the telechelic formation are (i) a prolonged heating (60 °C, 48 h); (ii) the optimal molar ratio of the reagents, PEG, and isocyanate 1 (1:4); and the use of hexane as the purification agent.

**Figure 2:**
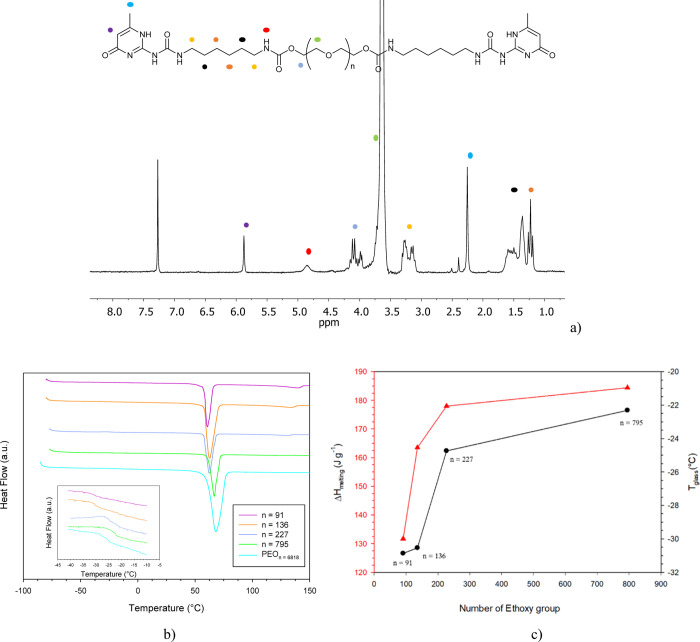
(a) ^1^H NMR spectrum (300 MHz, 25° C) of polymer 2 in CDCl_3_; (b) DSC thermograms of UPyPEG_*n*_UPy with different chain lengths compared to that of pure PEO_6818_ (300 KDa). The inset shows the *T*_g_ region; (c) melting enthalpy, Δ*H*_m,s_, and glass transition temperature, *T*_g_, for all the investigated UPyPEG_*n*_UPy samples.

The obtained telechelics were first characterized by high-resolution NMR and FTIR spectroscopies. Figure 2 shows a representative spectrum for the case of the UPyPEG_795_UPy sample.

As clearly shown in Figure 2a, the UPy end-capping of PEG moieties was successful. Specifically, complete difunctionalization was proven by integration of the unique proton resonances of the UPy moiety at ∼6 ppm and the unique proton resonances of the PEG moiety (namely, those of the CH_2_ groups in the repeating unit) at ∼ 3.5 ppm. Furthermore, the polymer purity is proved by the absence of unique proton resonances of the starting material 1 (*e.g.*, the signal of CH_2_ protons of the isocyanate moieties) (see the ^1^H NMR spectra reported in Figure S4). All the collected spectra of UPyPEG_*n*_UPy (namely, polymers 2–5 in Scheme 1) are fully consistent with the proposed structure for each investigated *n*, as shown in Figure S5. A comparison among them also clearly provides evidence of the increasing chain length of the PEG repeating units. FTIR spectra were also collected for each UPyPEG_*n*_UPy telechelic to further confirm the expected polymer structure. The spectra do not show the vibrational signal at ∼3500 cm^–1^ typical of the OH bond stretching, which is, in contrast, well evident in the case of pure PEG spectrum (Figure S6a–e). This further confirms the point that the above-described reaction took place through the foreseen mechanism.

In order to select the optimal UPy telechelic in terms of chain dynamics, measurements of DSC were carried out on all the synthesized polymers. PEG is a semicrystalline polymer with glass transition temperature, *T*_g_, well below room temperature, and this property is important to assure sufficient chain mobility and then self-repairing ability. Figure 2b compares the DSC traces of the UPyPEG_*n*_UPy networks at different *n* values. All the polymers show well-defined glass transition and melting phenomena, whose characteristic temperatures, *T*_g_ and *T*_m_, respectively, are influenced by the PEG molecular weight. As expected, both the glass transition temperature and the melting enthalpy increase with *n*, resulting in an enhancement of polymer stiffness (see Figure 2c). The UPyPEG_*n*_UPy crystalline fraction is obtained from the ratio between the experimental melting enthalpy, Δ*H*_m,s_, and the one expected for a fully crystalline sample, assumed to be Δ*H*_m_ = 206 J g^–1^.^36^ It increases from 60% to about 85% by increasing *n*, whereas the glass transition temperatures remain below −22 °C even in the case of PEG_795_ (*M*_W_ = 35 kDa). The thermograms also show another endothermal phenomenon between 120° and 150 °C, whose intensity decreases by increasing the PEG chain length. Such a broad signal is frequently observed in PEO-based systems and is assigned to the endothermic mixing between the crystalline PEG chain undergoing melting around 65 °C and the metastable liquid phase originating at the polymer glass transition.^37^

The conjunction of these two properties, namely, low *T*_g_ and the presence of crystalline domains, is therefore optimal to obtain polymers with high hydrogen bonding dynamics and good mechanical strength. Taking into accounts such results, we opted for UPyPEG_795_UPy (*M*_W_ = 35 kDa) as the SH UPy-telechelic unit for the blend component. Here, the amorphous phase is enough to allow the rearrangement of the polymer chains and to drive the healing of any cracks autonomously. At the same time, the crystalline phase contributes to the polymer’s mechanical properties helpful to contain the large fluctuations of the BP structure in the anode.^22^

Finally, thermogravimetric analysis showed that each investigated telechelic is very stable from a thermal point of view with degradation temperature exceeding at least 180 °C, independently on the PEG chain length (see Figure S7).

#### 3.1.2 SH UPyPEG_795_UPy—PEO Blend

The UPyPEG_*n*_UPy polymers have no optimal MW to form films with good free-standing properties. For this reason, the longer telechelic (UPyPEG_795_UPy) was selected to be homogeneously mixed with a higher MW polymer as PEO (300 kDa) to obtain a blend with a double function, namely, (i) SH capability ensured by the telechelic unit and (ii) good free-standing properties allowed by PEO. Specifically, three blends UPyPEG_795_UPy—PEO were prepared by mixing proper amounts of the single components to achieve the volume ratios (v–v) 40–60, 50–50, and 60–40. In the following, the samples will be labeled blend 40–60, blend 50–50, and blend 60–40, respectively (see Table 1). For the sake of clarity, here the first number refers to UPyPEG_795_UPy and the second one to PEO.

**Figure 3:**
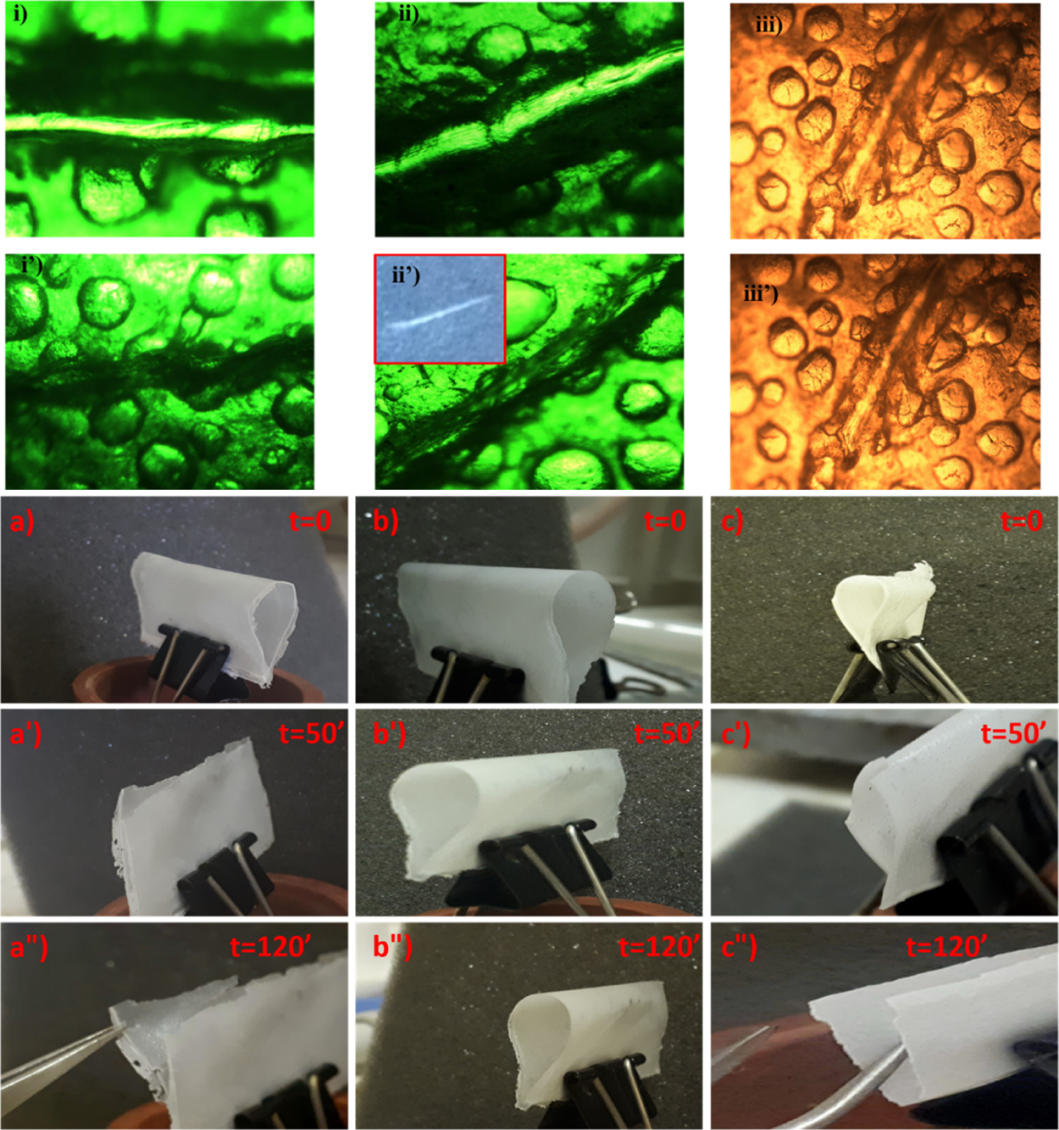
(Above) Optical microscopy images of the (UPyPEG_795_UPy)—PEO blends: 40–60 (i,i′), 50–50 (ii,ii′), and 60–40 (iii,iii′), just cut (above), and during the SH process (below). Bending test over time for the blends 40–60 (a,a′), 50–50 (b,b′), and 60–40 (c,c′).

Initially, the SH ability of the blends was qualitatively evaluated by scratching with a razorblade 100 μm-thick films, prepared as described in the Materials and Methods section, and following their spontaneous self-repairing upon time. Figure 3a shows the optical microscopy images obtained for all the investigated blends, immediately after cut (i, ii, iii) and after 2 h (i′, ii′, iii′). In the first two cases, the cracks are fully healed. In contrast, the SH of 60–40 is not recovered at all, as observed by comparing Figure 3iii,iii′. In order to exclude the SH effects due to the PEO component, the same test was also carried out on a pure PEO film, whose optical microscopy image is reported in Figure S8, showing that no crack is repaired even after 5 days of rest time.

The repairing ability was also checked in terms of the reconstruction of electric continuity. To this aim, a 50–50-based composite film was prepared including 15 vol % of conductive carbon (KS-10 Timcal). This film was connected to a multimeter and then cut in two pieces in order to follow the evolution with time of the film resistance. Figure S9 shows the gradual and full recovery of the composite resistance after the sample crack toward the initial value shown before the rupture.

In order to check the bending resistance of the blends, finally, the films were kept under forced folding for at least 2 h, as shown in Figure 3a–c. While the samples 40–60 and 60–40 underwent partial or complete rupture, 50–50 did not undergo fracture, remaining well flexible for about 4 h (Figures 3b,b′ and 8b).

These findings can be explained by the higher crystallinity observed in the 50–50 sample, resulting in an increased mechanical robustness, as proved by the good agreement of the experimental evidence coming from our multidisciplinary approach (DSC, ^13^C–^1^H solid-state NMR, and DMA), whose main results are summarized in Table 1.

**Figure 4:**
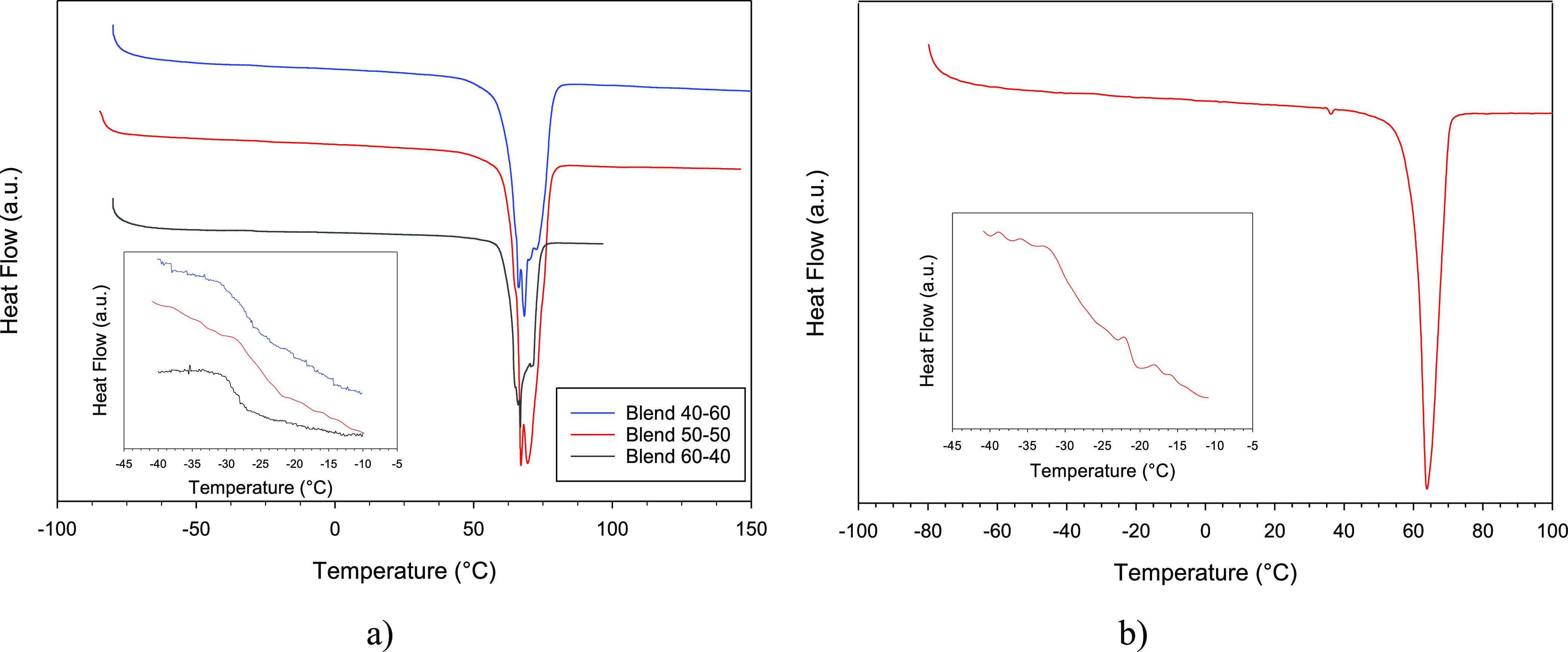
DSC thermograms of the (UPyPEG_795_UPy)—PEO blends 40–60, 50–50, and 60–40 (part a) and of the healed part of the blend 50–50 (see the inset in Figure 3ii′). The glass transition temperatures, *T*_g_, are highlighted in the figure insets.

Figure 4a compares the DSC thermograms of the three blends as prepared. Clear glass transition (see the inset) and melting phenomena are observed. While all blends exhibit a single *T*_g_, the melting endotherms are well structured, suggesting a physical mixing of multiple phases with slightly different melting temperatures and enthalpies. Indeed, all the blends exhibit *T*_g_ and Δ*H*_m,s_ lower than those of pure UPyPEG_795_UPy and PEO components, resulting in semicrystalline systems where the amorphous fraction is more extended and less viscous at ambient temperature. However, 50–50 exhibits melting enthalpies of 10% and 20% higher than 40–60 and 60–40, respectively, which suggests a larger fraction of crystalline domains. As the melting enthalpy of 100% crystalline UPyPEG_*n*_UPy is not known, it is not possible to determine the amount of the crystalline fraction from DSC data, and just the trend should be considered.

Figure 4b reports the DSC plot of the healed part of the 50–50 sample. Here, the several melting peaks undergo coalescence, and the glass transition temperature, *T*_g_ < −34 °C, significantly decreases. Both these phenomena may be related to the formation of new dynamic H-bonds driven by the approaching of the UPy-terminated chains, which physically link the two components into a unique and highly dynamic network. The repaired damage also indicates that the presence of crystalline PEG domains does not interrupt the dynamic hydrogen bonding formation and the related properties.

As for the pure components, the blends are also thermally stable at least up to 250 °C, as shown by the corresponding TGA plots, reported in Figure S10.

**Figure 5:**
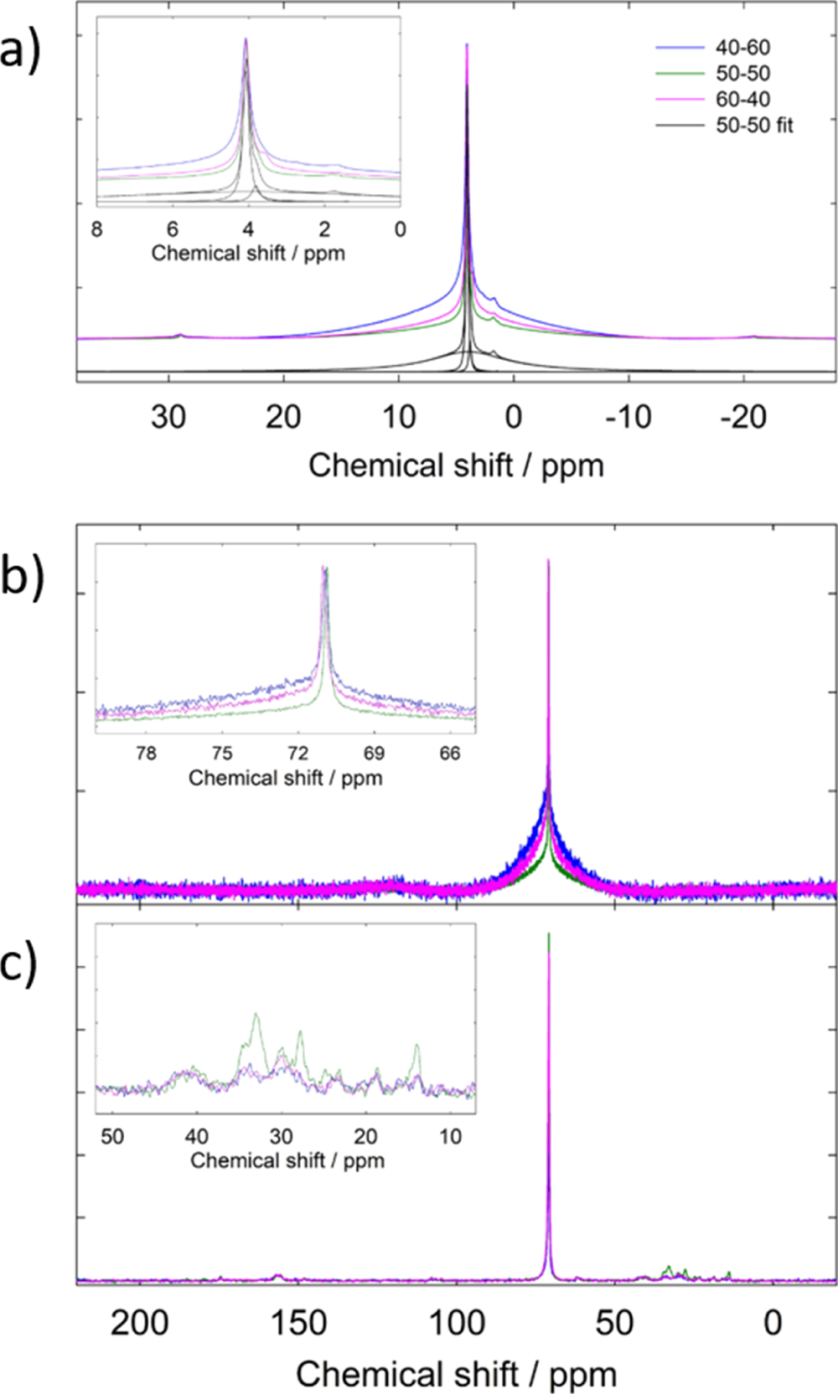
(a) ^1^H spectra for the pristine blend 40–60 (blue), blend 50–50 (green), and blend 60–40 (pink) compositions; (b) ^13^C spectra for the pristine blend 40–60 (blue), blend 50–50 (green), and blend 60–40 (pink) compositions; and (c) ^1^H–^13^C CPMAS spectra for the blend 40–60 (blue), blend 50–50 (green), and blend 60–40 (pink) compositions.

The blends were also investigated by means of solid-state NMR spectroscopy. Figure 5a shows the ^1^H one-pulse spectra, which exhibit narrow signals at ∼4.1 ppm, overlapped to broader bands centered at the same chemical shift. Smaller features can be observed at ∼3.8 ppm and in the range of 1.9–1.3 ppm. The main peak at 4.1 ppm can be associated with the protons of the PEO units −(CH_2_–CH_2_–O)– present both in the PEO and UPy chains, in good agreement with the attribution reported for the molecule in solution (Figure 2a), and the narrow and broad contributions can be attributed to signals from the crystalline and amorphous polymer strands, respectively.^37,38^ The small feature in the 3.8–3.5 ppm range, which shifts to lower fields with increasing PEO content, can be associated with the −CH_2_– moieties in the UPy-based polymer. Finally, the small features in the 2.5–1.3 region can be associated with the terminal methyl groups of the polymer chains, again in good agreement with the attribution reported in Figure 2a.^38,39^ The remaining protons of the UPy unit could not be observed because of their low concentration. Although the main peaks near 4 ppm are very similar, a closer inspection shows some changes with the membrane composition (see the inset of Figure 5a). The main part of the spectrum can be fitted in terms of three Gaussian contributions, two of them centered at 4.1 ppm and the third one ranging in the range 3.8–3.5 ppm, depending on the composition. As previously stated, this third component is associated with the CH_2_– moieties of the UPy-based part of the polymer. The two components at 4.1 ppm, in turn, can be attributed to system crystalline and amorphous fractions.^38,39^ The best fit performed on the base of this simple model shows that the amorphous/crystalline fraction does not vary linearly with the composition, but the 50–50 sample has the highest content of crystalline domains (see Table 1). This trend has been qualitatively confirmed by the same analysis performed on the ^1^H-decoupled ^13^C spectra (Figure 5b) on the sharp and broad resonances observed for all the three samples at 71 ppm. Finally, ^13^C–^1^H CPMAS spectra, reported in Figure 5c, show the same main resonance at 71 ppm due to the −(CH_2_–CH_2_–O)– moieties in the polymer chains and smaller features associated with the amide (∼175 ppm), aromatic rings (152–165 ppm), and aliphatic portions (10–50 ppm) (see Figure 5c and the inset).^40^ Again, all the spectra are very similar. However, upon normalization to the main resonance, it is possible to observe that the 50–50 sample has a better signal-to-noise ratio than the other two compositions, which is well evident in the aliphatic portion of the spectrum (see the inset). This calls for a higher cross-polarization efficiency, which can be due to different factors, that is, the lower mobility of the interested fragments if the cross-polarization is associated with intrachain mechanisms and/or to the shorter distance among fragments if associated with the interchain ones. Both these mechanisms are compatible with higher crystallinity of this sample, which is associated with a closer packing of the polymeric chains, in agreement with the results obtained from the DSC and DMA analyses.

**Figure 6:**
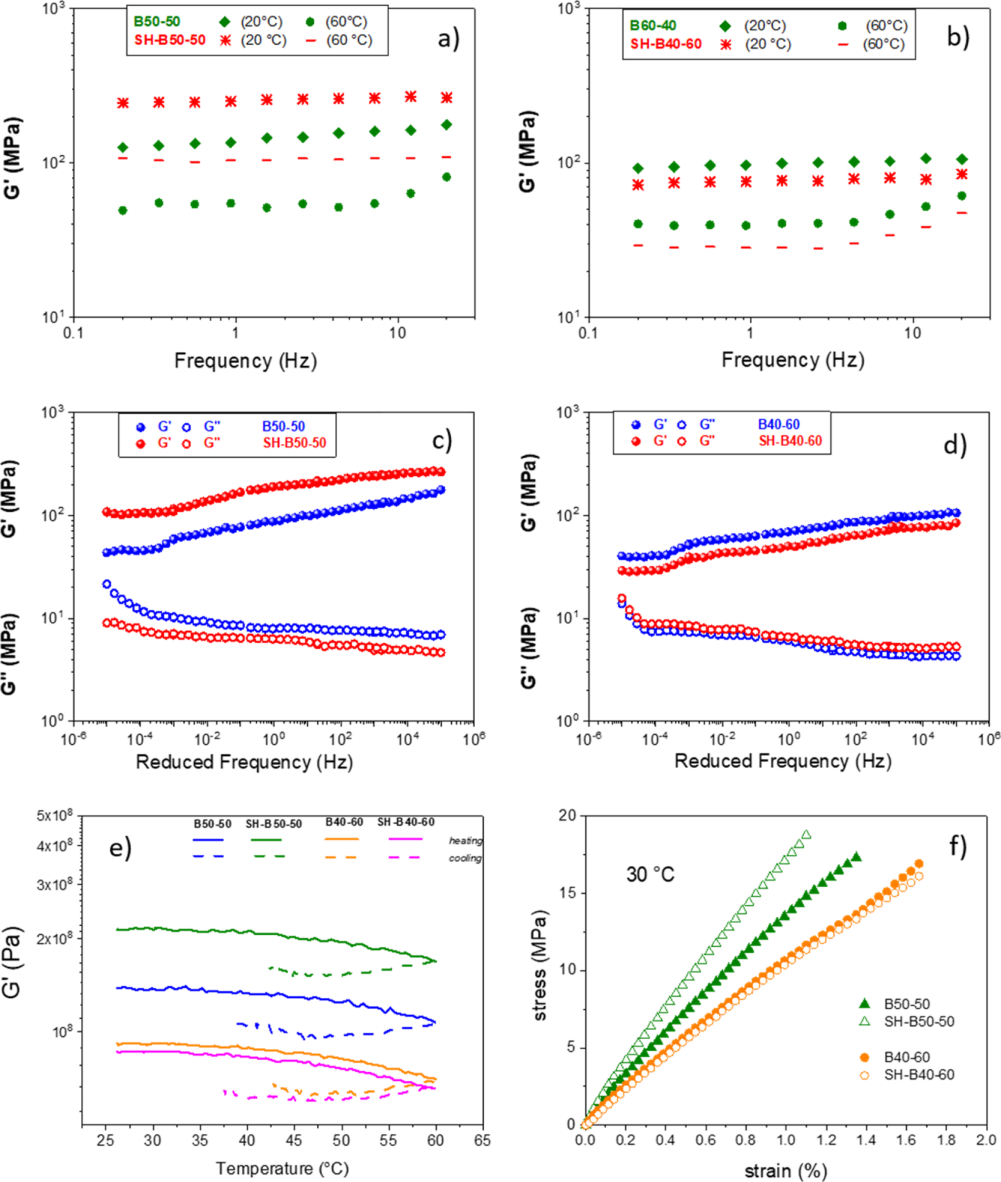
(a,b) Frequency sweep tests, at 20 and 60 °C, for the (UPyPEG_795_UPy)—PEO blends: (a) blend 50–50 both pristine B50–50 and self-healed SH-B50–50 and (b) blend 40–60 both pristine B40–60 and self-healed SH-B40–60. (c,d) Master curves of storage (*G*′) and loss (*G*″) moduli reduced at a reference temperature of 40 °C: (c) blend 50–50 both pristine B50–50 and self-healed SH-B50–50; (d) blend 40–60, both pristine B40–60, and self-healed SH-B40–60. (e) Temperature evolution of the storage moduli (*G*′) from 25 to 60 °C performed on pristine blend 50–50 (B50–50), self-healed blend (SH-B50–50), blend 40–60 (B40–60), and self-healed blend SH-B40–60. (f) Stress–strain plot for blend 50–50, both pristine B50–50 and self-healed SH-B50–50; and blend 40–60, both pristine B40–60 and self-healed SH-B40–60.

DMA was used to investigate the mechanical properties of 50–50 and 40–60 blends. The 60–40 sample was not considered due to nonefficient SH capability. Figure 6 shows the frequency dependence of the storage (*G*′) and loss (*G*″) elastic moduli for the 50–50 (a) and 40–60 (b) samples, at 20 and 60 °C, both pristine (B50–50 and B40–60) and restored (SH-B 50–50 and SH B40–60). In the whole temperature range, all the samples reveal storage modulus higher than the loss one, which highlights their good elasticity. Nonetheless, DMA analysis underlines some crucial differences between the samples:

(i) The *G*′ moduli of 50–50 is 127 MPa at 20 °C, which is roughly 30% higher than that of 40–60 gel (96 MPa), thus implying greater mechanical strength of the former blend, in agreement with the higher crystallinity revealed by DSC and NMR;
(ii) The self-healed 50–50 sample exhibits higher elastic modulus than the pristine film. This is likely due to a larger number of strong HBs between the two components (UPyPEG_795_UPy and PEO) taking place in such a blend;
(iii) *G*′ decreases upon heating (while *G*″ clearly increases) due to the gradual weakening of the polymer structure. There is also a slight dependence on the frequency of both modules for some samples, with a reduction in the *G*′/*G*″ ratio below 10, which is typical of “weak gel”-like systems.^41^

With regard to this last point, a different behavior is observed in the case of self-healed 50–50 blend, which is able to maintain the mechanical properties of a “strong gel”-like system (both modules independent from ω and with a *G*′/*G*″ ratio >10) even at relatively high temperature (*i.e.*, 60 °C).^42^ In a nutshell, the SH process enhances the mechanical strength as well as the thermostability of the 50:50 blend.

Master curves were constructed by using the “time–temperature superposition (TTS)” principle, based on the Williams–Landel–Ferry model.^43^ This allows the prediction of the mechanical behavior of polymers as a function of frequency over time scales that are not experimentally accessible.^44,45^ For each sample, the frequency sweeps acquired at five test temperatures (from 20 to 60 °C every 10 °C) were shifted to the reference temperature of 40 °C, allowing to extend the frequency window from 0.01 mHz to 0.1 MHz (Figure 6c,d). In such a wide frequency range, all the membranes show an elastic solid-like behavior since the elastic module *G*′ is averagely 10 times larger than the viscous module *G*″. However, both the two 40–60 membranes and the pristine 50–50 one show module values approaching toward the low-frequency region, which is an indication of a weakening of the polymeric film. Accordingly, a clear loss of elasticity occurs to the systems as a consequence of a “*strong–weak*” network transition. Despite this, no crossover between the moduli was found in the whole frequency range, implying the absence of “*gel*–*sol*” transition. This indicates that the blends possess excellent stability over a wide range of time scales, either in undamaged or in self-healed state. These master curves also confirm the exceptional mechanical properties of the 50–50 restored membrane, which maintains a strong gel behavior on a wide range of time scales.

The temperature sweep tests on a heating–cooling cycle are shown in Figure 6e, where the temperature behavior of the storage modulus (*G*′), in the range 25–60 °C on the first heating and cooling scan cycle, is displayed. All the samples exhibit a similar behavior: *G*′ slightly decreases during heating, but it is almost completely recovered on cooling. Actually, a certain hysteresis is observed, which means that more time, less than 1 h, is needed to fully recover the module to its initial value in cooling. Indeed, the mechanical spectra executed on more thermal cycles are practically superimposable (see Figure S11), clearly indicating the thermoreversibility of these systems.

Finally, Figure 6f shows the stress–strain behavior of the pristine and self-healed 50–50 and 40–60 membranes up to the limit of their respective linearity region. Undamaged blends exhibit similar tensile stress (*ca.* 17.5 MPa), but the elongation at yield point is higher for 40–60 (1.66%) than for 50–50 (1.33%). Such higher elasticity is likely due to the rubber-like properties of the network. As can be clearly seen, both the healed polymer films can effectively self-recover to their original strain after a healing duration of 24 h at room temperature. It is worth noting that the self-healed 50–50 polymer can sustain a remarkably higher tensile stress, that is, *ca.* 22 MPa, than the undamaged membrane, *ca.* 17 MPa. Such a massive increase in membrane strength is clearly compatible with the formation of an efficient hydrogen-bonded crosslinked structure.

### 3.2 Battery Cycling

To the best of our knowledge, contrary to the silicon-based anodes, chemical SH of BP-based anodes for LIBs or SIBs has not been explored yet. As described in previous reports, the huge volume expansion (up to 300%) observed in this materials upon cycling is typically managed by means of BP composites with high amount of carbon, which acts as a volume buffer. Such a strategy is in principle promising but causes a decrease of the volumetric energy density and the success depends on several variables, including the type of carbon, anode composition, electrolyte, and additives.^3^

This work describes, for the first time, the use of a novel binder with SH ability in the aqueous processing of BP anodes for SIBs. To this aim, the blend 50–50 (namely, UPyPEG_795_UPy—PEO 50–50 volume ratio) was selected as the SH polymeric component of the electrode due to its excellent elastic properties and recovering ability. To properly evaluate the repairing effect of such a binder on the electrochemical behavior of BP in SIBs, the functional properties of the anode were compared with those observed for the same anode including a conventional CMC–PAA binder. The galvanostatic behavior was evaluated on coin cells from 0.01 and 2 V *versus* Na^+^/Na at different current densities by using sodium as the counterelectrode.

**Figure 7:**
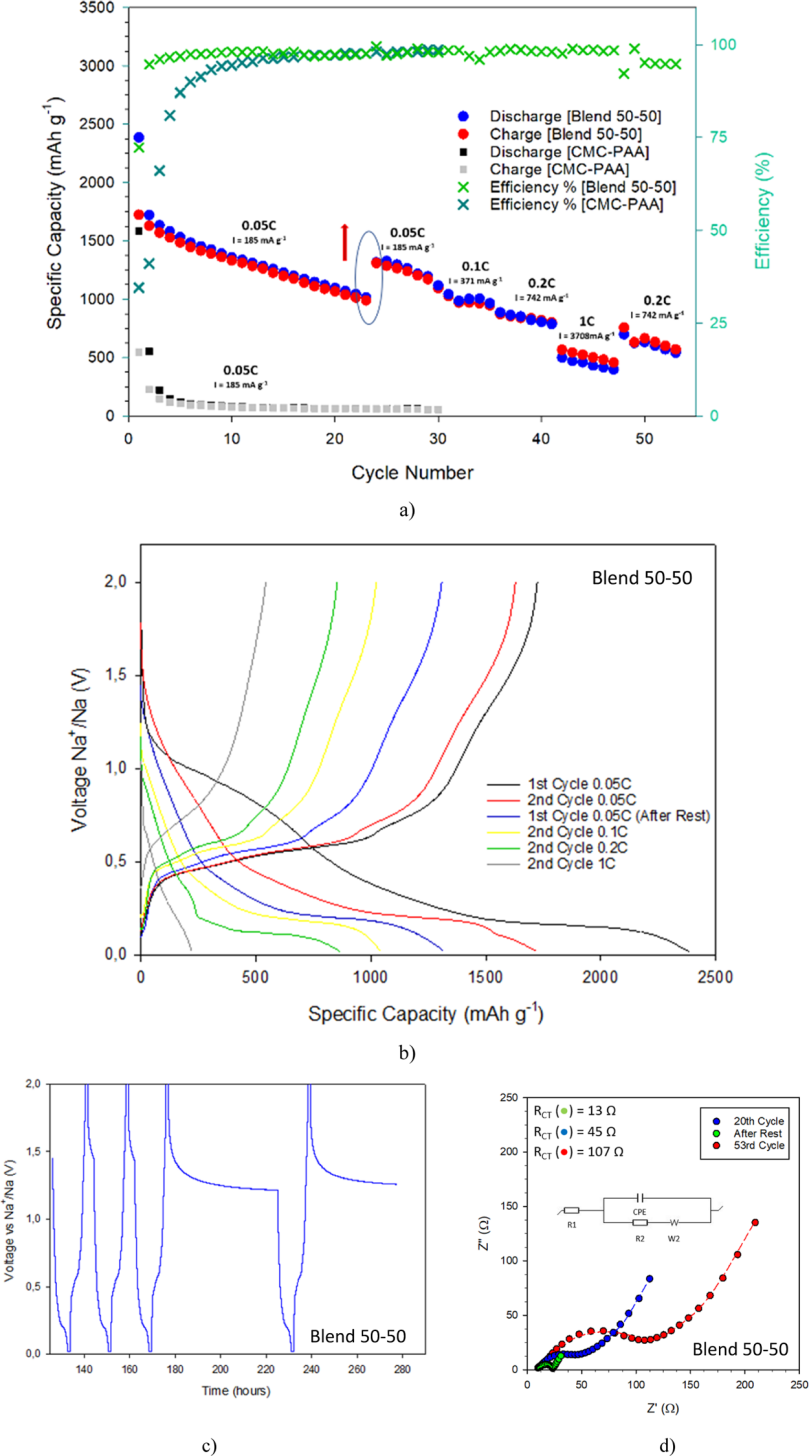
(a) Comparison of the rate performances of two BP anodes (0.05 C: *I* = 185 mA g^–1^; 0.1C: *I* = 371 mA g^–1^; 0.2C: *I* = 742 mA g^–1^; and 1C: 3708 mA g^–1^), including the SH binder (circles, blue: discharge; red: charge; and light green: efficiency) and the conventional CMC–PAA (squares, black: discharge; gray: charge; and dark green: efficiency), (b) voltage profiles of the SH BP anode, (c) voltage profiles upon time at C/20 or 185 mA g^–1^ including a rest period of 48 h, and (d) Nyquist plots collected during and at the end of the galvanostatic cycling tests on the SH BP anode.

Figure 7a,b reports the rate performances and the corresponding voltage profiles of the BP anodes made with the SH and the CMC–PAA binders. The BP mass loading in the anode was 1.26 and 1.6 mg cm^–2^, respectively. Electrodes including different BP amounts were also investigated, namely, 2.5 mg cm^–2^ for B50–50 and 1 mg cm^–2^, but no significant differences were observed in the electrochemical performances (Figure S12 ad ref (6)). On cycling, the first cycle discharge (desodiation) capacity is 2450 mA h g^–1^ at 0.18 A g^–1^ (corresponding to a cycling rate of C/20 based on the theoretical capacity of BP 1C = 2596 mA h g^–13^), very close to the theoretical one for both the anodes. Upon sodiation, both the samples show a typical stepwise process related to the formation of Na_*x*_P species, followed by a pronounced plateau at around 0.3 V *versus* Na metal, due to the final Na_3_P formation. After the first cycle, the discharge capacity of SH anodes decreases up to 1700 mA h g^–1^, as typically observed in these systems. However, starting from the second cycle, the cycling stability of the two electrodes is dramatically different. After 25 cycles at the same current density (0.185 A g^–1^), the BP anode with a 50–50 binder retained ∼70% of the initial capacity, proving the SH agent ability to assure stronger adhesion among the phosphorus particles, thus mitigating structural instability. In contrast, the capacity of the anode with a CMC–PAA binder abruptly drops to about 100 mA h g^–1^ after the first five cycles, corresponding to a capacity retention less than 10%, in agreement with what is generally observed for pure BP anodes.^6,46^

The impressive stabilizing effects of the 50–50 SH binder is further confirmed when this anode is kept to rest for 48 h (see Figure 7a,c). Contrary to the anode with CMC–PAA, after the cycling breaks, the SH anode regains about 80% of the capacity lost during the first 25 cycles at 0.185 A h g^–1^ (increasing from 1000 mA h g^–1^ to 1500 mA h g^–1^). This demonstrates the maintenance of a good electric contact upon cycling, which is secured by the autonomic healing of the mechanical cracks through the UPy-driven multiple hydrogen bonding. The repairing ability of the binder also affects the electrode cycling performances. In fact, as the current density increases, the specific capacity only slightly decreases, achieving 1000 and 850 mA h g^–1^ at 0.371 A g^–1^ (C/10) and 0.742 A g^–1^(C/5), respectively, with Coulombic efficiency very close to unit. Although a definite plateau in the capacity behavior is not reached, as observed in the case of SH pure Si anodes,^18,22^ the binder exhibits an impressive stabilizing effect even at high current densities. In fact, the SH BP anode still works well around 500 mA h g^–1^. This result is better than those observed in the case of other mitigation strategies, for instance, the introduction of Ge into BP to enhance the elastic softness, for which similar capacity retention was only observed at a 4 times lower current density.^46^

EIS was used to check the SH effect on the interfacial resistances (see Figure 7d). The spectra were obtained at the 20th cycle (i) before, (ii) after the 48 h rest period, and (iii) at the end of the cycling test. The charge transfer resistance is in principle very low in both cases and undergoes further decrease in consequence of the rest from 45 to 15 Ω. This trend indicates the growth on the anode of a stable, thin, and low-resistance SEI, which is able to protect the BP surface avoiding abrupt capacity decays.

In the case of CMC–PAA-based BP anode, charge transfer resistances much higher than those measured for the SH electrode were obtained by impedance spectroscopy measurements (Figure S13) after electrochemical cycling, namely, 1840 and 1221 Ω, respectively. The spectrum at the end of the cycling test appears more complex, likely because of the formation of one or more different passivation layers, reasonably thicker than what, in contrast, noticed in the B50–50 anode.

**Figure 8:**
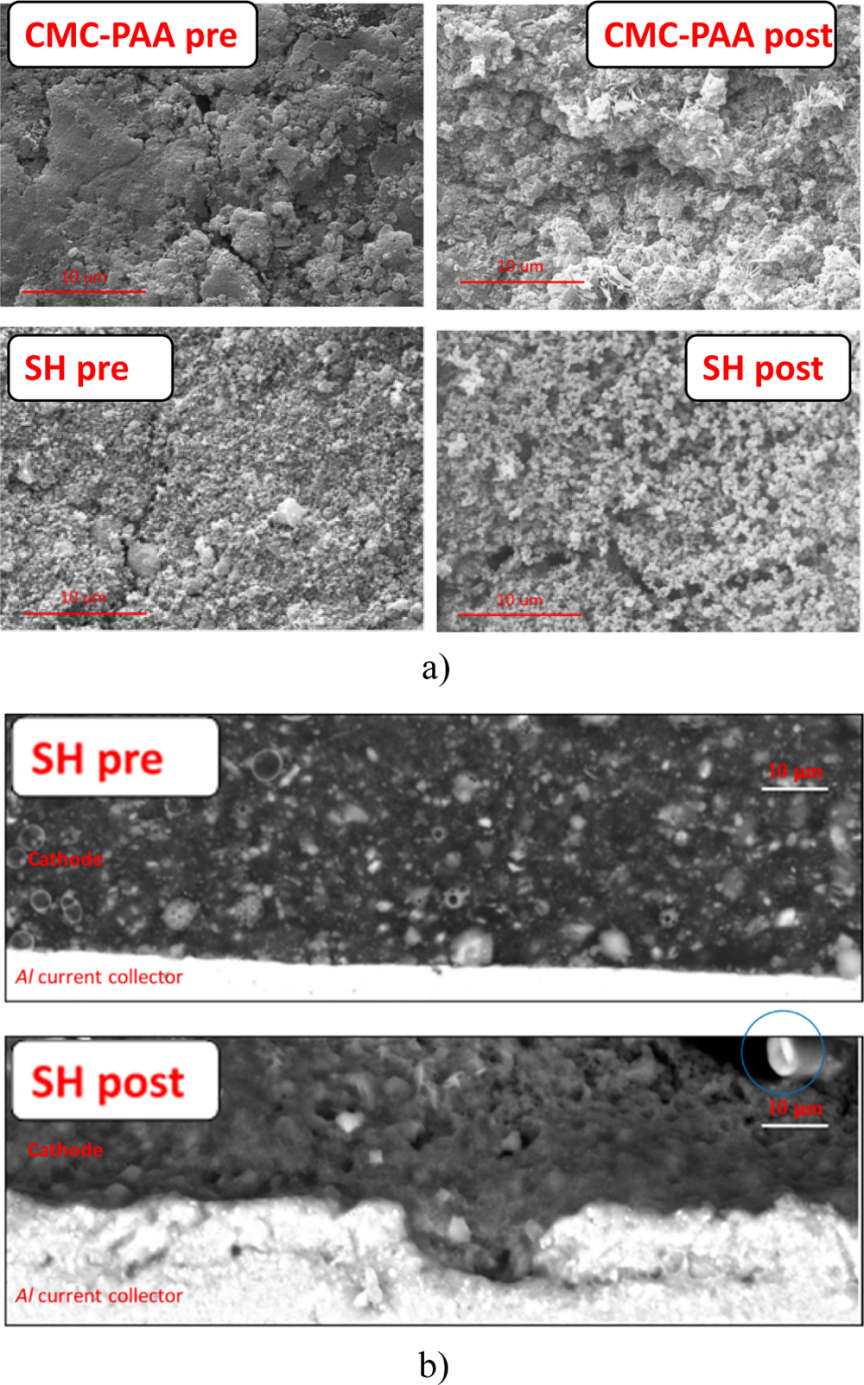
(a) Top-view images of the CMC–PAA-based (up) and SH-based (down) BP anode before and after cycling; (b) cross-sectional SEM images of the pristine SH-based BP anode (up), and of the same electrode observed after the end of galvanostatic cycling tests (down).

Finally, both top-view and cross-sectional scanning electron microscopy (SEM) images (Figure 8) were collected on the cell before the galvanostatic cycling and at the end of the whole tests (after 55 cycles at different current densities). The top-view images (part a) provide evidence that the SH anode displays a much more flat and smooth morphology with respect to the CMC–PAA-based electrode, which shows even a much discontinuous surface. This low rugosity is also maintained after electrochemical cycling, where no significant increase of roughness is noted. A texture of small grains distributed on the surface is also present reasonably related to the SEI formation and byproducts of electrolyte decomposition. Higher magnification of the SEM top-view images is included in Figure S14 of the Supporting Information. The cross-sectional images (part b) show that the BP anode including the SH binder is able to preserve the electrode morphology. The cross-section of the pristine anode (a) reveals a slightly porous electrode with a thickness of about 35 μm and a homogeneous distribution of the particle sizes. Despite such low porosity, the Na migration along the anode is still ensured thanks to a polymer chain-assisted mechanism, allowed by the low *T*_g_-PEG-based SH binder, further plasticized by the carbonate liquid electrolytes. Except for a slight increase of porosity, the cycled electrode does not reveal any significant structural degradation or reduction of adhesion with the current collector, which is still very good, contrary to what is observed in the case of CMC–PAA binders where post-mortem SEM-FIB/EDX revealed serious coating layer detachments from the Al foil, as already shown in our previous paper.^6^ In addition, the cycled electrode thickness does not increase, confirming that such a binder prevents the BP anode from irreversible volume expansion thanks to its SH ability.

## 4 Conclusions

For the first time to the best of our knowledge, we have reported on a novel and sustainable polymeric blend with SH ability as a binder in BP-based anodes for SIBs. The repairing reactivity is intrinsic and based on dynamic multiple hydrogen bonding enabled by UPy-telechelic networks. Specifically, we have successfully demonstrated that the SH properties of the binder have remarkable beneficial effects on both cycling performances and stability of the electrode in Na-ion cells.

Although additional work is necessary to meet the commercial requirements of SIBs or LIBs, undoubtedly the SH performances of this binder are very promising and will be further explored. In particular, our results lay the groundwork to extend the use of the UPy-telechelic backbones also to other electrochemically active materials suffering huge volumetric changes, even in synergy with other strategies which already proved their ability to improve the structural stability, for example, carbon as a buffering agent.

## Supporting Information Available

Raman Spectra, SEM, and XRD of BP; ^1^H NMR of compound 1 and polymer 2; ^1^H NMR of polymers 2–5 and ^13^C NMR of polymer 4,5; FTIR spectra of pure PEG and polymers 2–5; TGA of polymers 2–5; image of optical microscopy of crack on pure PEO and photo of blend 40–50 after 4 h of bending test; electric SH test; TGA of the investigated blends; additional DMA analysis of the blend 50–50 and 40–60; galvanostatic cycling of SH BP anode at different mass loadings; Nyquist plots of SH BP anode before cycling and of CMC–PAA-based BP anode both before and after cycling; and SEM top view images of both SH and CMC–PAA BP anode before and after cycling tests (PDF)
