# Supplementary material for: Autonomous Self-Healing Strategy for Stable Sodium-Ion Battery: A Case Study of Black Phosphorus Anodes
Source: ACS Appl Mater Interfaces. 2021 Mar 15;13(11):13170–82. doi: 10.1021/acsami.0c22464 (PMC8041259; doi:10.1021/acsami.0c22464)
Supplement: Supplementary file 1 — am0c22464_si_001.pdf [file am0c22464_si_001.pdf]

Supporting Information for:

Autonomous self-healing strategy for stable Sodium-ion battery: a case study of Black Phosphorus anode

D. Callegari [a±], S. Colombi [a±], A. Nitti [a], C. Simari [b,d], I. Nicotera [b,d], C. Ferrara [c,d],

P. Mustarelli [c,d], D. Pasini [a*],and E. Quartarone [a,d*]

[a] Department of Chemistry, University of Pavia, Via Taramelli 16, 27100 Pavia, Italy

[b] Department of Chemistry and Chemical Technologies, Università della Calabria, Via Pietro Bucci, 87036 Arcavacata di Rende, Cs Italy

[c] Department of Materials Science, University of Milano Bicocca, Via Cozzi 55, 20125 Milano, Italy

[d] National Reference Centre for Electrochemical Energy Storage (GISEL) - INSTM, Via G. Giusti 9, 50121 Firenze (ITALY)

[±] Equal contributions

*Corresponding Author: dario.pasini@unipv.it

*Corresponding author: Eliana.quartarone@unipv.it

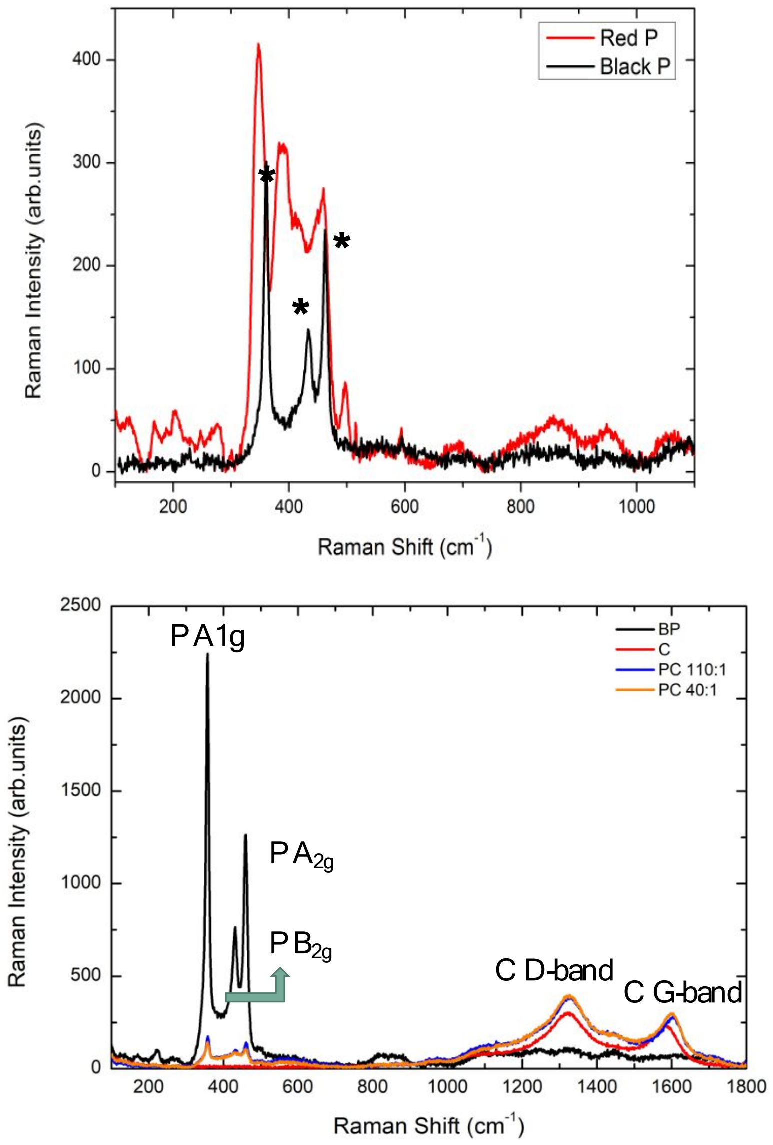

Fig. S1: Raman spectra of: **(up)** Black Phosphorus, obtained by high energy ball milling starting from Red Phosphorus (whose corresponding spectrum is the red line) at 500 rpm and milling ball /RP mass ratio of 40/1 for 2 hours; the stars show the typical A_{1g}, B_{2g}, A_{2g} modes for orthorhombic BP; **(down)** Black Phosphorus including carbon as used to prepare the anode slurry (see the spectra of sample 40:1). Here the spectra of pure BPs and pure Carbon have been added for a sake of comparison.

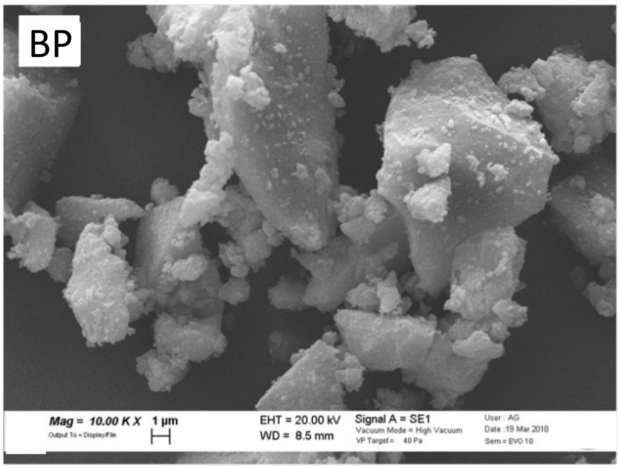

Fig. S2: Sem image of Black Phosphorus (BP), obtained by high energy ball milling starting from Red Phosphorus (whose corresponding spectrum is the red line) at 500 rpm and milling ball /RP mass ratio of 40/1 for 2 hours. The image of the BP-C used in the anode is discussed in ref. 6. Figure reproduced with permission from ref. 29 Copyright 2019 American Chemical Society

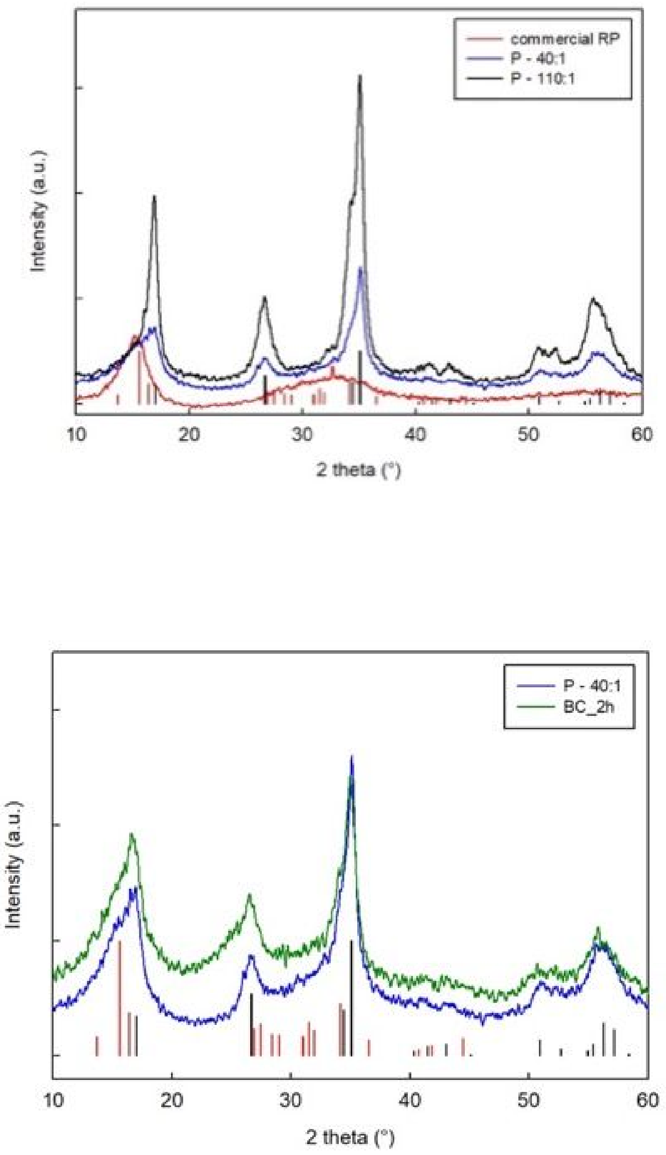

Fig. S3: XRD patterns of: **(up)** Black Phosphorus, obtained by high energy ball milling starting from Red Phosphorus (whose corresponding spectrum is the red line) at 500 rpm and milling ball /RP mass ratio of 40/1 and 110/1; **(down)** Black Phosphorus including carbon, in the composition as used to prepare the anode slurry. Vertical red and black lines refer to the Bragg intensity related to red Phosphorus (RP) and black Phosphorus (BP) as reported by PDF cards 44-0906 and 76-1958, respectively. Figure reproduced with permission from ref. 6 Copyright 2020 IOP Publishing.

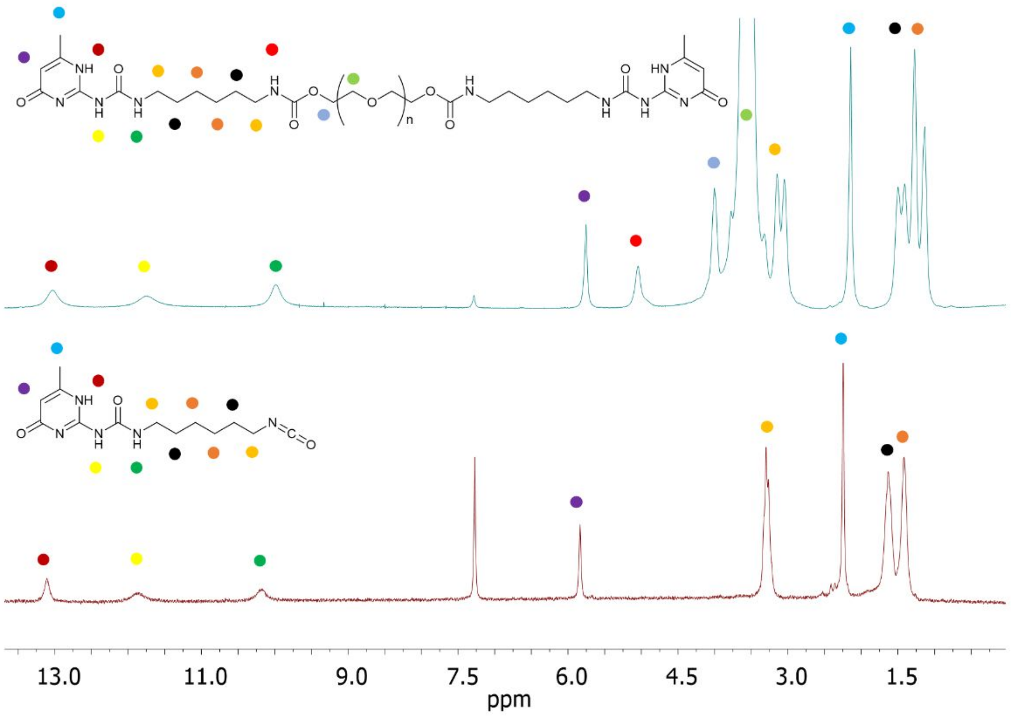

Figure S4. Comparison between the ^{1}H NMR spectra (300 MHz, 25°C) of reagent 1 and purified polymer 2.

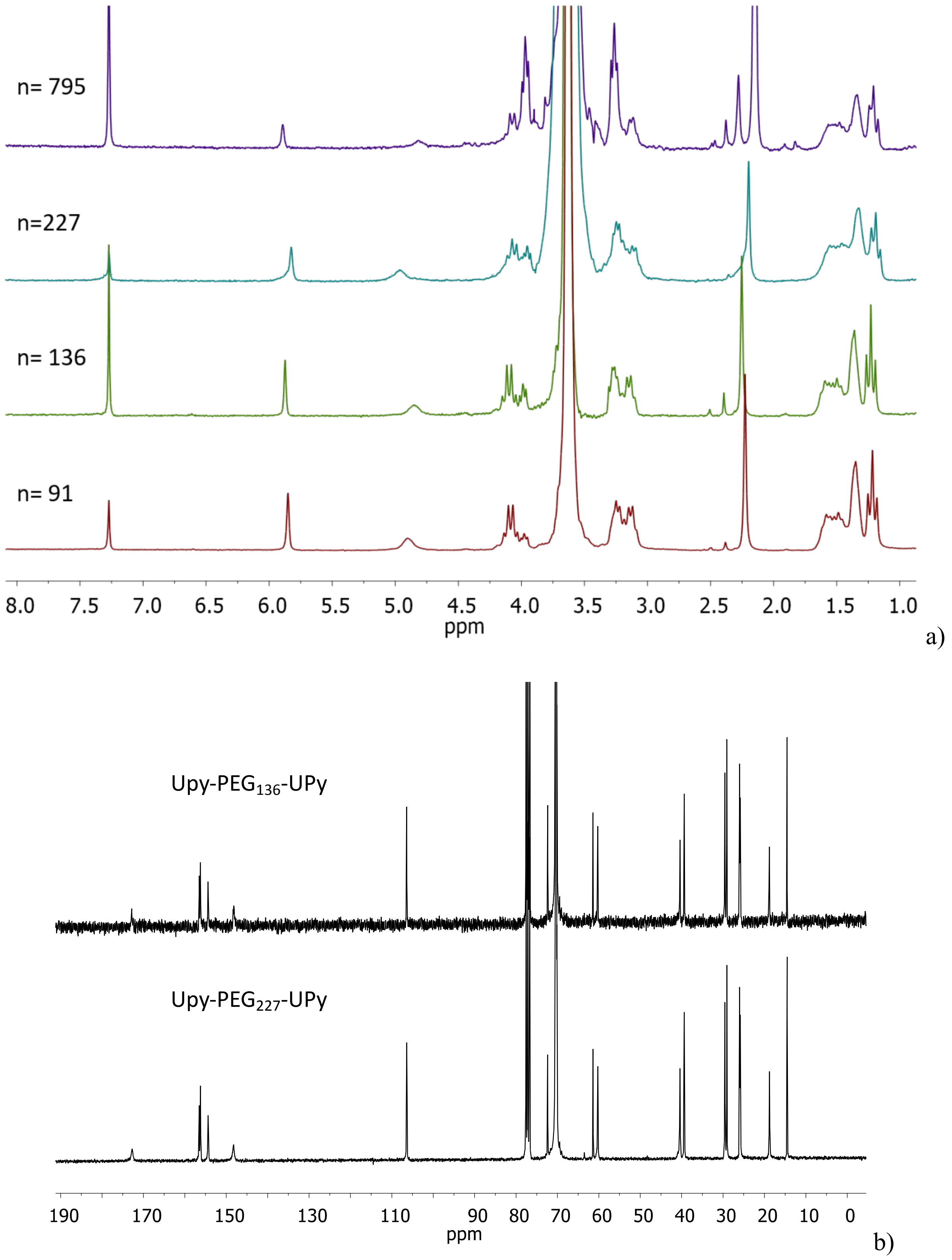

Figure S5. Comparison between A) the 1H NMR spectra (200 MHz, 25°C) of purified polymers 2-5 A) and B) the ^{13}C NMR spectra of purified polymers 3-4.

a)

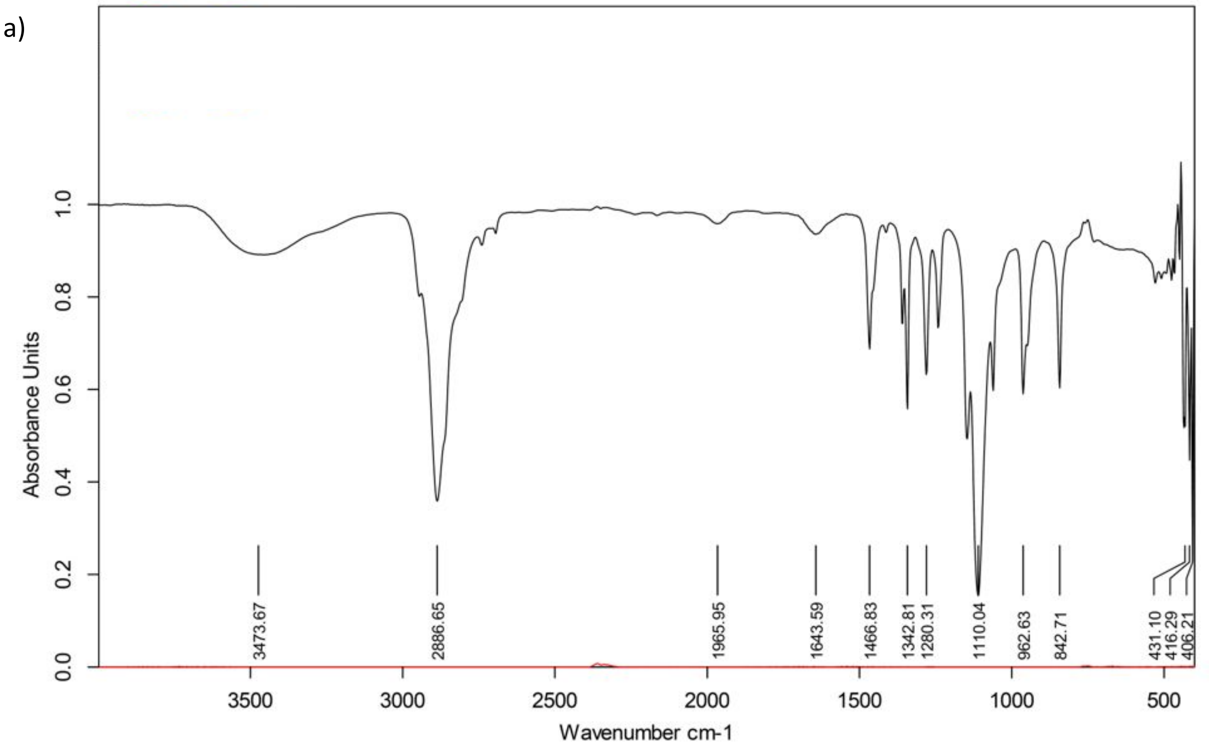
Absorbance Units
Wavenumber cm-1
3473.67
2886.65
1965.95
1643.59
1466.83
1342.81
1280.31
1110.04
962.63
842.71
431.10
416.29
406.21

b)

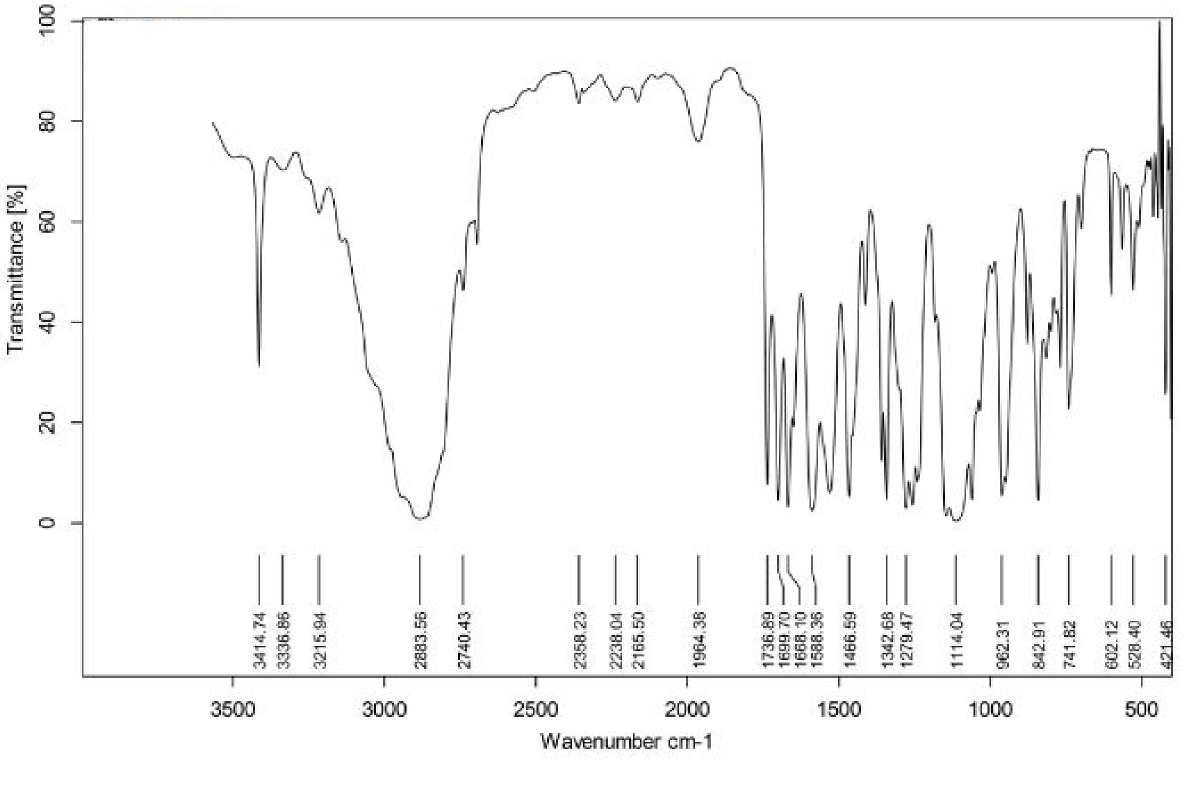
Transmittance [%]
Wavenumber cm-1
3414.74
3336.86
3215.94
2883.56
2740.43
2358.23
2238.04
2165.50
1964.38
1736.89
1699.70
1668.10
1588.36
1466.59
1342.68
1279.47
1114.04
962.31
842.91
741.82
602.12
528.40
421.46

c)

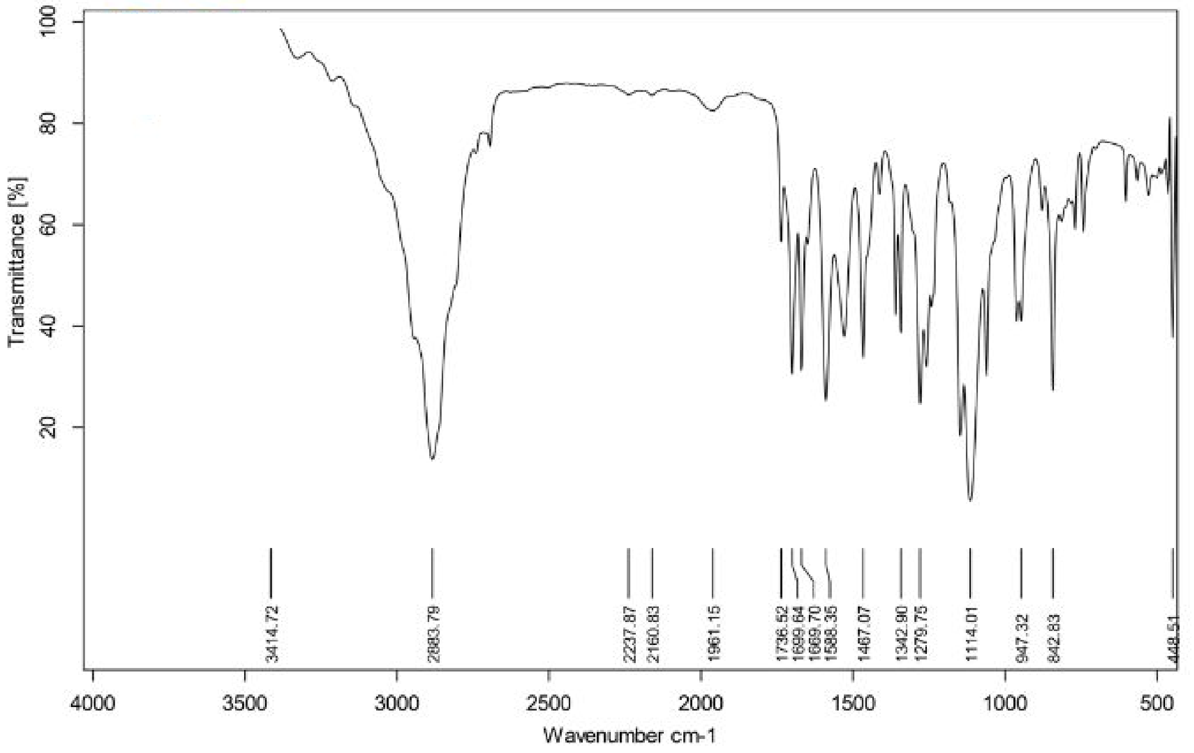
Transmittance [%]
Wavenumber cm-1
3414.72
2883.79
2237.87
2160.83
1961.15
1736.52
1699.64
1669.70
1588.35
1467.07
1342.90
1279.75
1114.01
947.32
842.83
448.51

d)

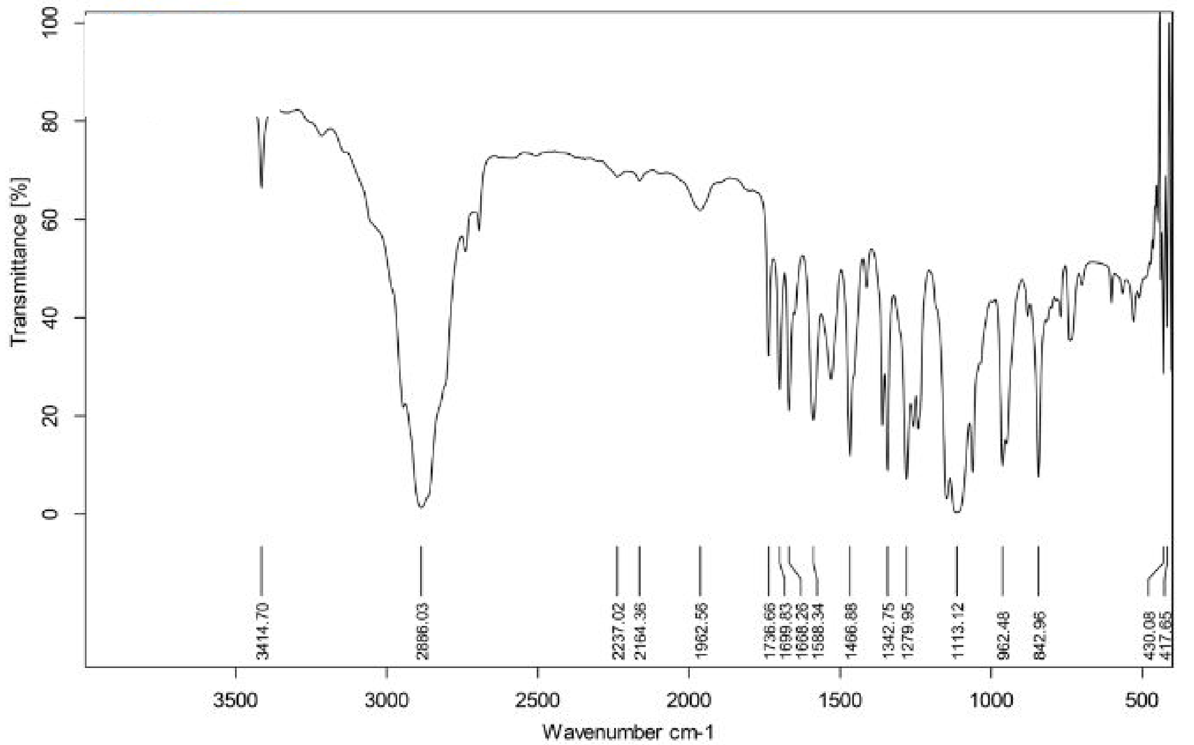
Transmittance [%]
Wavenumber cm-1
3414.70
2886.03
2237.02
2164.36
1962.56
1736.66
1699.83
1668.28
1588.34
1466.88
1342.75
1279.95
1113.12
962.48
842.96
430.08
417.65

e)

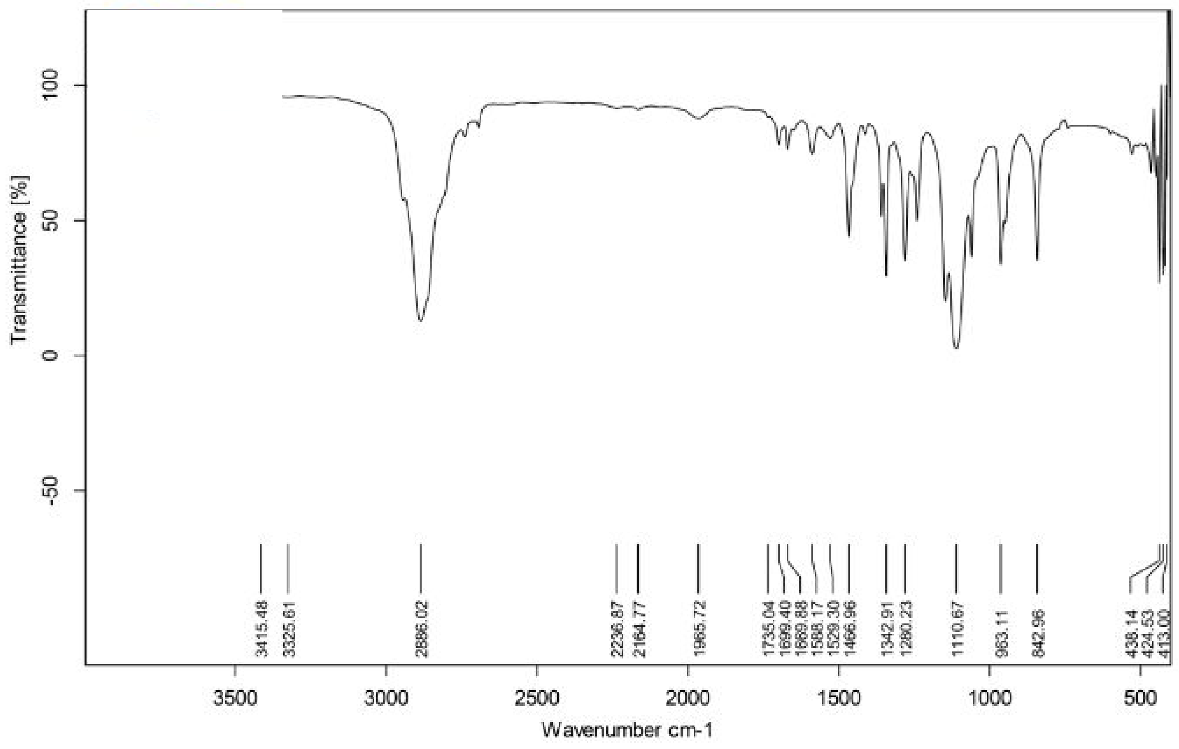

Figure S6. IR spectra of pure PEG_{227} (MW=10000 Da) (a) and purified polymers 2, 3, 4 and 5 (respectively b, c, d and e)

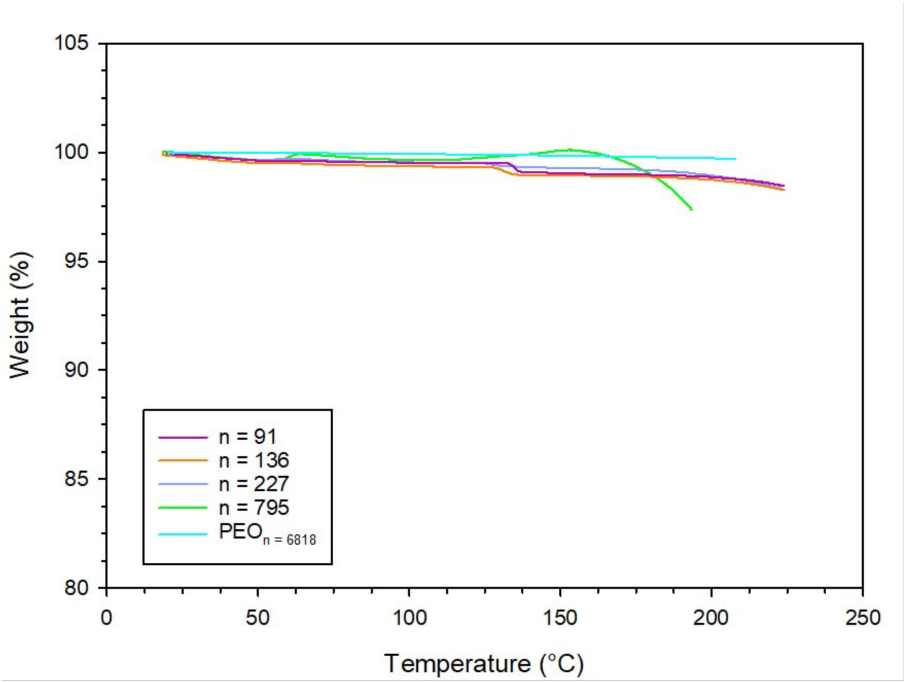

Figure S7. TGA plots of all the investigated UPyPEG$_n$UPy telechelics. The thermogram of pure PEO (300 kDa) is also reported as a comparison.

a)

b)

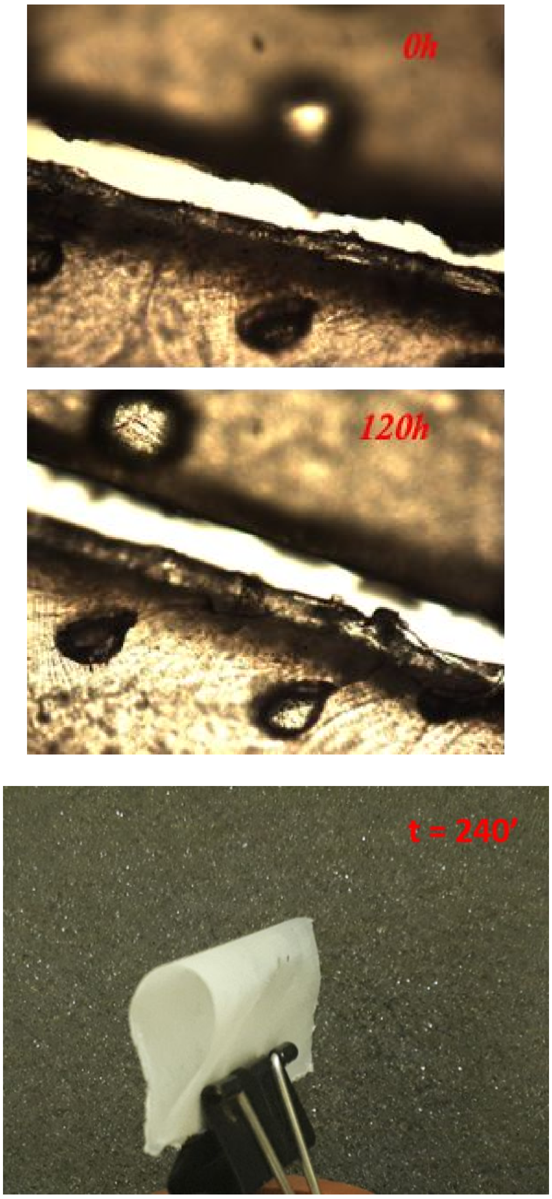

Figure S8. a) Optical micrographs of a PEO (MW=300 KDa) film, as cut and after 120 hours of rest time and b) 4 hours Bending test for the blends 50-50

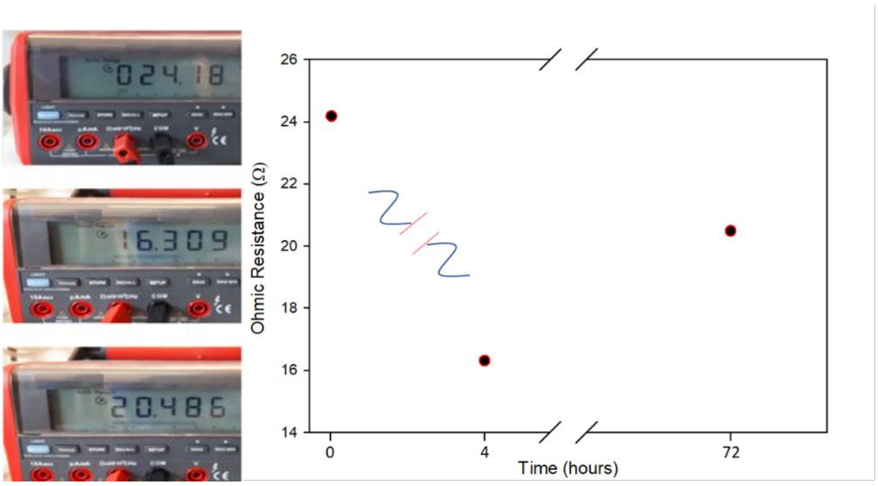

Figure S9. Electric resistance measurements of a composite film based on the B50-50 blend and carbon (15 vol% of Graphite KS 10), before the damage and during the recovering time. The blue and red lines is an illustration of the absence of electric contact caused by the cut.

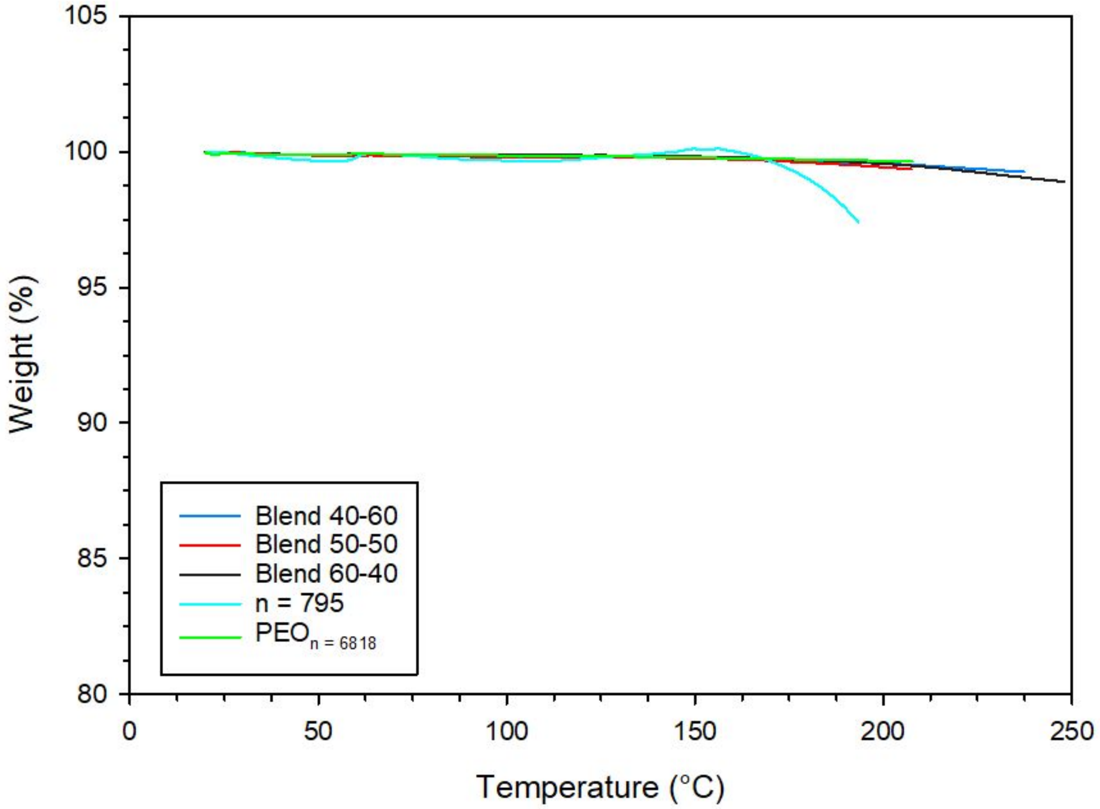

Figure S10. TGA plots of all the investigated $UPyPEG_{795}UPy$ - PEO blends. The thermogram of $UPyPEG_{795}UPy$ and pure PEO is also reported as a comparison.

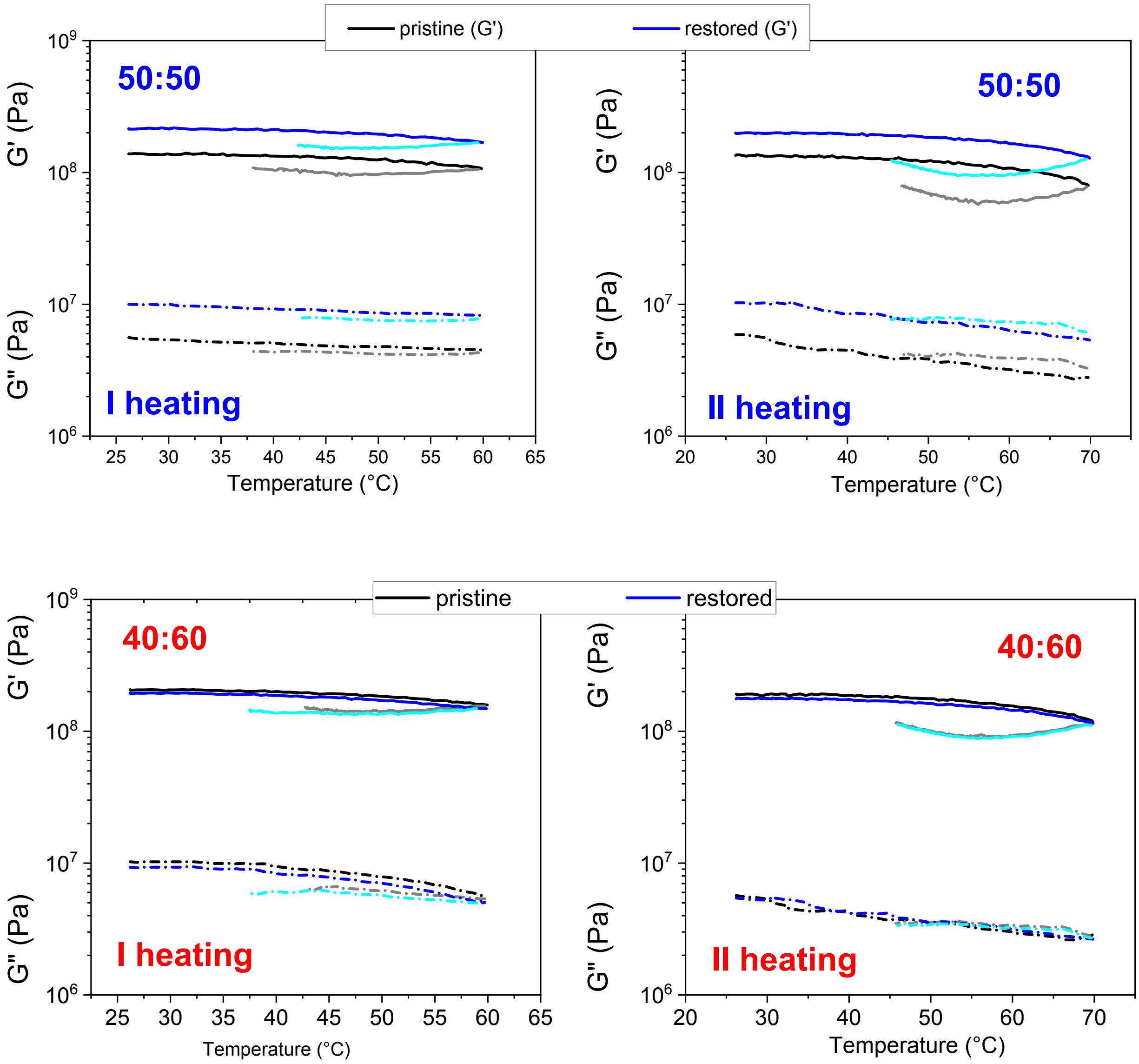

Figure S11. Frequency sweep tests carried out on more thermal cycles from 25 °C to 60 °C, performed on 50-50, 50-50_restored (self-healed), 40-60 and 40-60_restored (self-healed) membranes.

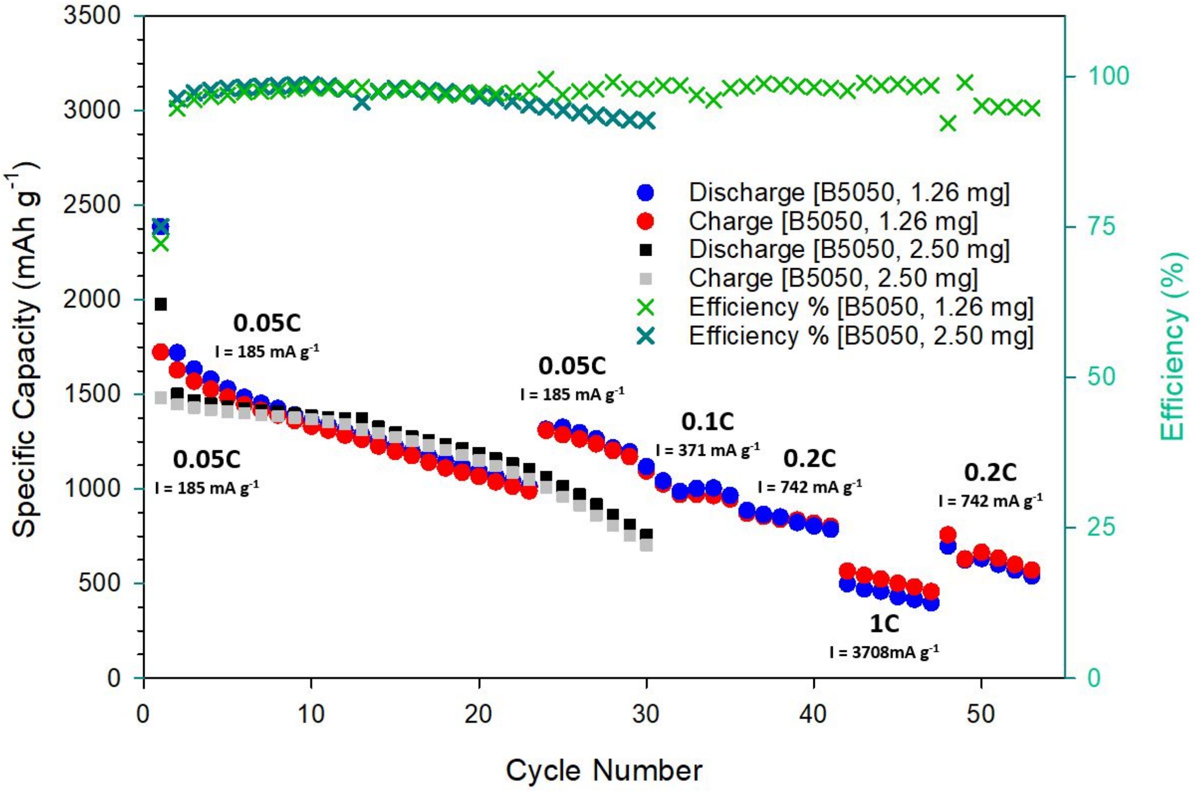

Figure S12. Comparison of the rate performances of two BP anodes, including the self-healing binder with a mass loading of 1.26 mg cm^{-2} (circles, blue: discharge, red: charge; light green: efficiency) and of 2.50 mg cm^{-2} (squares, black: discharge, grey: charge; dark green: efficiency).

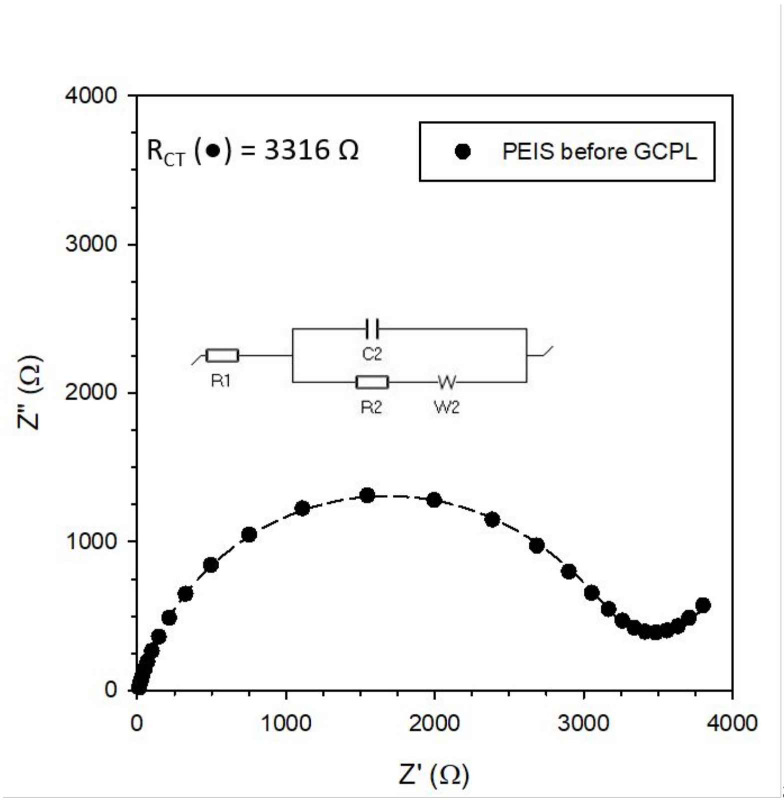

a)

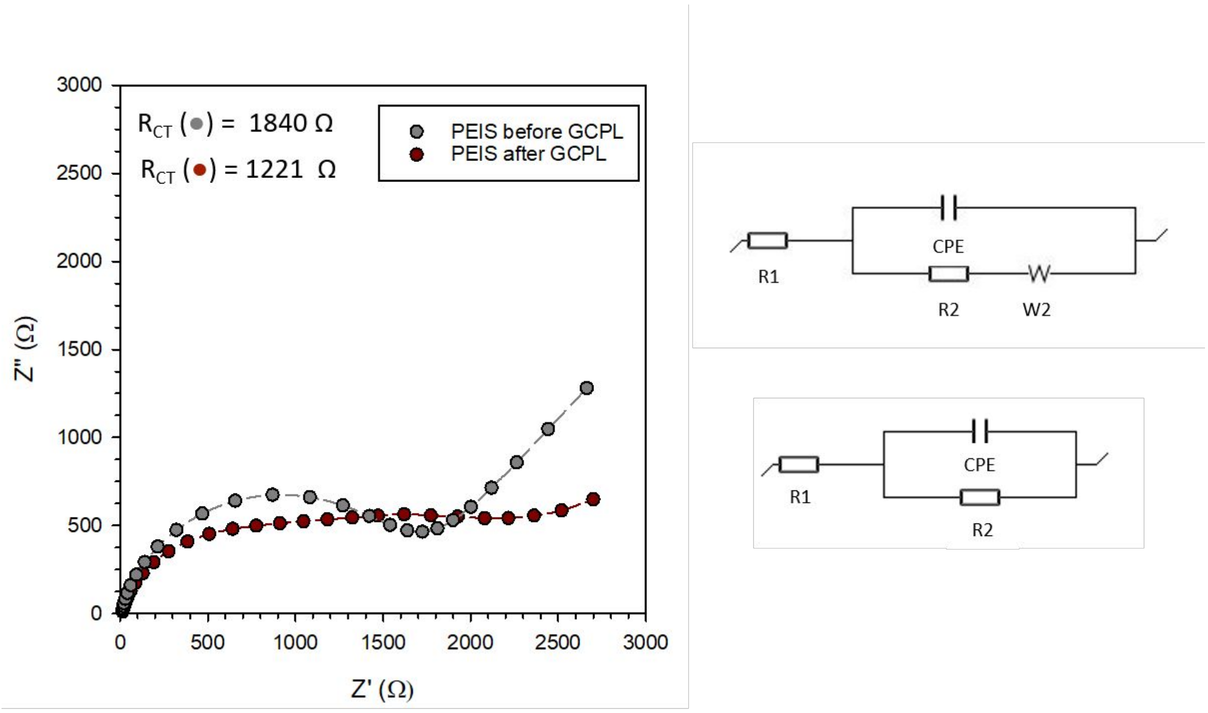

b)

Figure S13. Nyquist plots collected a) on the SH BP anode (before the galvanostatic cycling tests) and b) on the CMC-PAA BP anode (before and at the end of the galvanostatic cycling tests)

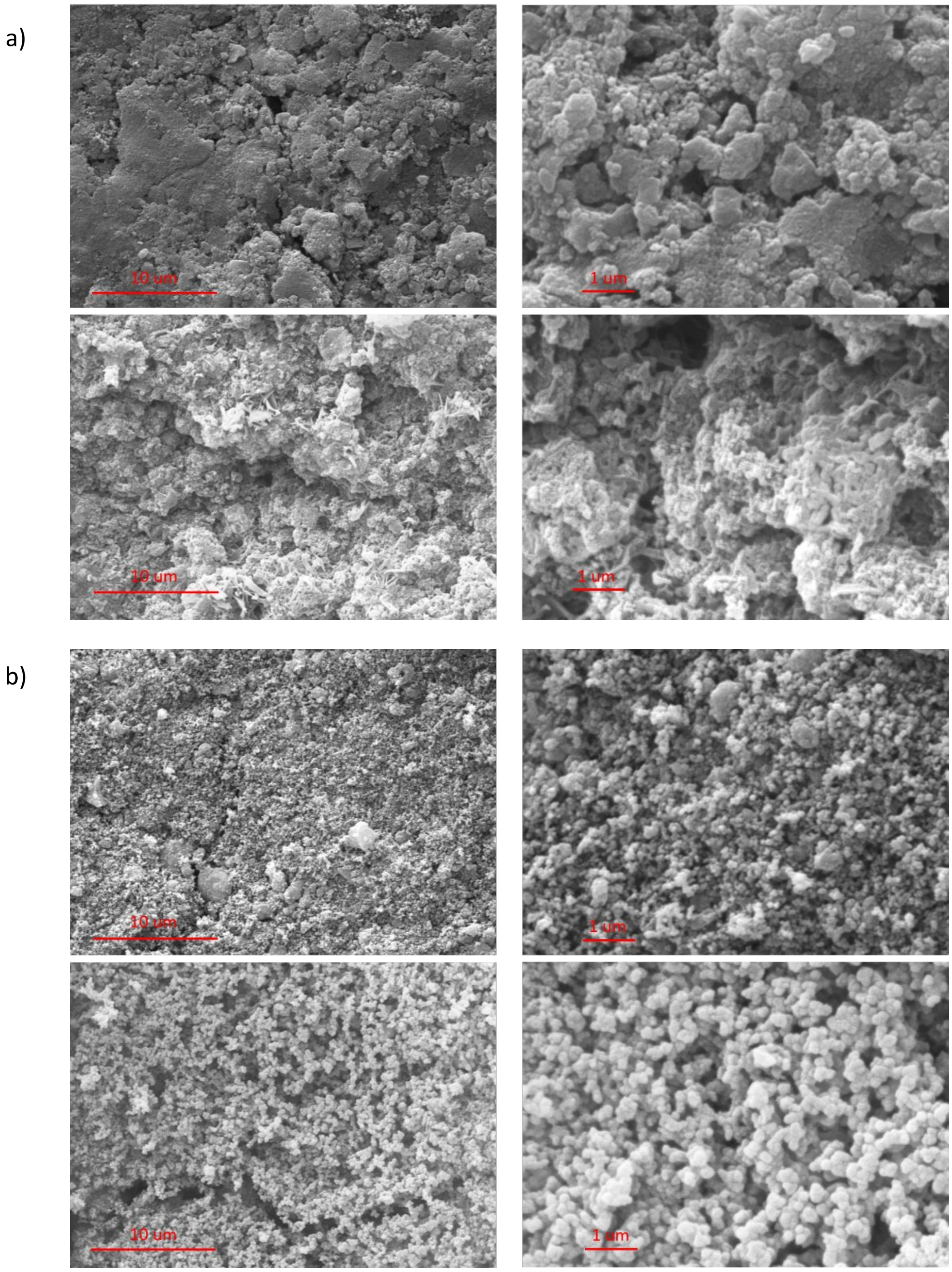

Figure S14. SEM images at two different magnification (5 kx: left and 20 kx: right) of a) anode with CMC-PAA binder and b) anode with blend 5050 binder. (pristine: up and after galvanostatic cycling test: down)
